# Integration of Laccase-like Nanozymes and Sensing Strategies for Food Quality and Safety Monitoring

**DOI:** 10.3390/foods15183309

**Published:** 2026-09-18

**Authors:** Man Zhou, Tian Jiang, Chenglin Li, Chunhua Dai

**Affiliations:** School of Food Science and Engineering, Jiangsu University, Zhenjiang 212013, China; manzhou@ujs.edu.cn (M.Z.);

**Keywords:** laccase-like nanozymes, sensing strategies, food quality, food safety

## Abstract

Laccase-like nanozymes (LLNs) are a class of functional nanomaterials inspired by natural laccases. Due to their excellent catalytic performance, structural tunability, and superior stability, LLNs have emerged as promising tools for food quality and safety monitoring. This review establishes a critical structure–activity–application framework for LLNs, correlating bioinspired and structural engineering strategies, with the regulation of active sites, electronic structures, and catalytic microenvironments, and, ultimately, with their analytical performance in food matrices. Key insights are provided into how LLNs are integrated with colorimetric, fluorometric, electrochemical, and multimodal sensing platforms. Furthermore, particular emphasis is placed on their applications in food quality monitoring, including the detection of bioactive markers, freshness monitoring of meat products, authentication and adulteration analysis of tea, and assessment of other quality-relevant parameters. For food safety, the sensitive detection of mycotoxins, pesticide and antibiotic residues, and food additives is specifically highlighted. Despite these advances, challenges remain in elucidating catalytic mechanisms and active-site structures, establishing standardized evaluation criteria, and improving selectivity and reliability in complex food matrices. Future research should therefore focus on rational active-site engineering, in situ mechanistic characterization, standardized performance evaluation, and the integration of LLNs with multi-mode, portable, and intelligent sensing technologies to facilitate practical food analysis.

## 1. Introduction

Laccases (benzenediol:oxygen oxidoreductases, EC 1.10.3.2) are multicopper oxidases that catalyze the oxidation of phenolic substrates [1,2]. Laccases constitute a diverse class of enzymes, rather than a single enzyme. Although their multicopper active-site architecture is generally conserved, source-dependent variations in the surrounding protein shell among fungal, bacterial, plant, and insect laccases can influence their substrate preferences and optimal pH and temperature ranges. During catalysis, laccases use molecular oxygen (O_2_) as the terminal electron acceptor and reduce it to water, without requiring exogenous H_2_O_2_ as an oxidant [3,4]. Laccases exhibit broad substrate specificity and can oxidize various aromatic compounds containing hydroxyl, amine, carboxyl, and methoxy groups and their derivatives [2]. The green catalytic profile and their broad substrate specificity have made them valuable in various fields, including sensing, food, biomedicine, environmental remediation, and others [4,5]. However, the practical applications of natural laccases are often limited by their sensitivity to environmental conditions, high production costs, and difficulties in recovery and reuse [6,7,8]. Industrial laccases are mainly obtained from microbial sources, particularly fungi and bacteria, owing to their rapid growth and relative ease of extraction and purification. Their production generally involves microbial cultivation or fermentation and subsequent enzyme extraction and purification, with the overall cost being affected by production yield and downstream processing [4].

The emergence of nanozymes, nanomaterials with intrinsic enzyme-like activities, has opened new avenues for developing robust, scalable, and efficient biocatalytic alternatives [9,10]. Among these, laccase-like nanozymes (LLNs) have attracted considerable interest due to their ability to replicate the catalytic function of natural laccase while offering enhanced stability, tunable activity, and ease of functionalization. Since the first report of copper-containing carbon dots (Cu-CDs) with laccase-like activity in 2015 [11], the family of LLNs has rapidly expanded to include coordination polymers [12], metal-organic frameworks (MOFs) [13,14], single-atom nanozymes (SAzymes) [15], and composite nanomaterials [16]. In contrast to natural laccases, which are restricted to protein scaffolds, LLNs can be constructed from diverse materials via versatile synthesis and structural-regulation strategies, conferring broad compositional and structural tunability [2]. Although LLNs may offer advantages in stability, reusability, and scalable manufacture, their actual manufacturing costs depend on precursor selection, synthesis complexity, batch-to-batch reproducibility, and the feasibility of large-scale production [9,17,18]. Therefore, LLNs cannot yet be considered universally less expensive than natural laccases.

The field of LLNs is currently undergoing accelerated development. Through structural mimicry of the multicopper active-site architecture inherent to natural laccases, numerous LLNs have been rationally designed using Cu-containing nanomaterials [19,20]. These copper-based LLNs have demonstrated marked potential by virtue of their high catalytic efficiency and tunable reactivity, which render them suitable for applications across the environment, medical, and other industrial sectors [18,21]. In parallel, additional metal centers, such as Mn, Fe, Ag, Co, and Ce, can also manifest laccase-like functions, showing unprecedented catalytic mechanisms [22,23,24,25,26]. Currently, existing reviews on LLNs mainly focus on the copper-based nanozymes [18,21] and their analytical applications in the fields of environment and biomedicine [17,27]. However, systematic reviews that specifically focus on the application of both copper-based and non-copper-based LLNs in food quality and safety assurance remain limited. LLNs combine laccase-like catalytic activity with the advantages of nanomaterials, including high structural stability, tunable catalytic properties, and the flexibility to regulate their active sites and surface properties, making them promising candidates for sensitive and robust food quality and safety monitoring. Therefore, a comprehensive review is needed to bridge this gap, consolidate current knowledge, and provide insights into the relationship between LLNs structure, catalytic properties, and analytical performance in food applications.

**Figure 1:**
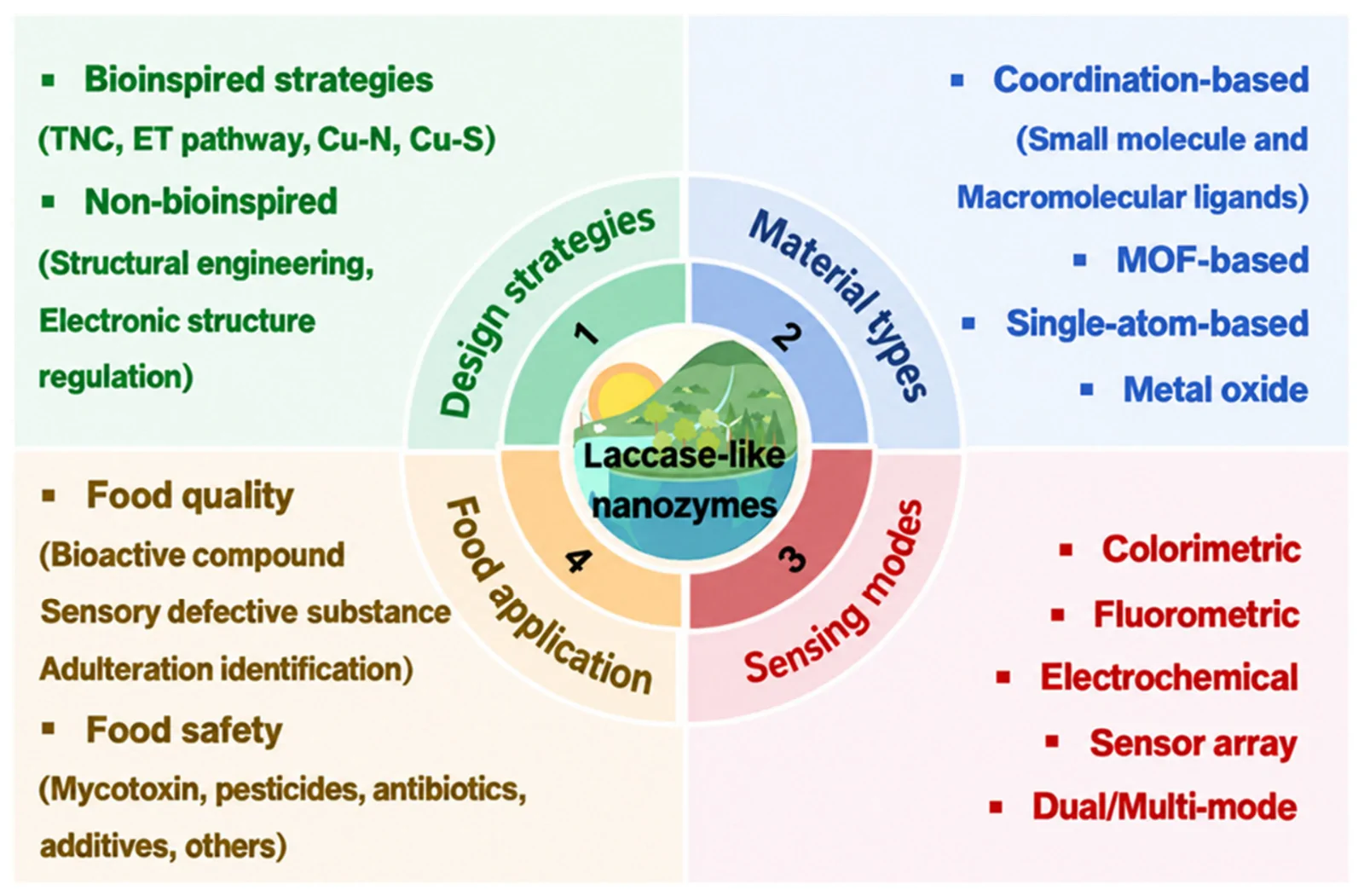
Schematic overview of this work.

This review emphasizes the development of LLNs for analytical applications in the food industry (Figure 1). The structural basis of LLNs is elucidated, with emphasis on the design strategies for enhancing their performance and on a detailed classification of material types based on structural features. Furthermore, the sensing platforms based on LLNs, ranging from conventional colorimetric, fluorescent and electrochemical methods to innovative fluorescence and multimodal sensor arrays, are explored. Specifically, the applications of LLNs in conjunction with sensing technologies are elaborately introduced, emphasizing food quality assessment (e.g., bioactive compounds, sensory defective substances, food adulteration) and food safety monitoring (e.g., mycotoxin, pesticides, antibiotics, and food additives). Finally, challenges and future perspectives are outlined to advance the applications of LLNs in the food sector.

## 2. Design Strategies and Material Types for LLNs

### 2.1. Design Strategies for LLNs

**Figure 2:**
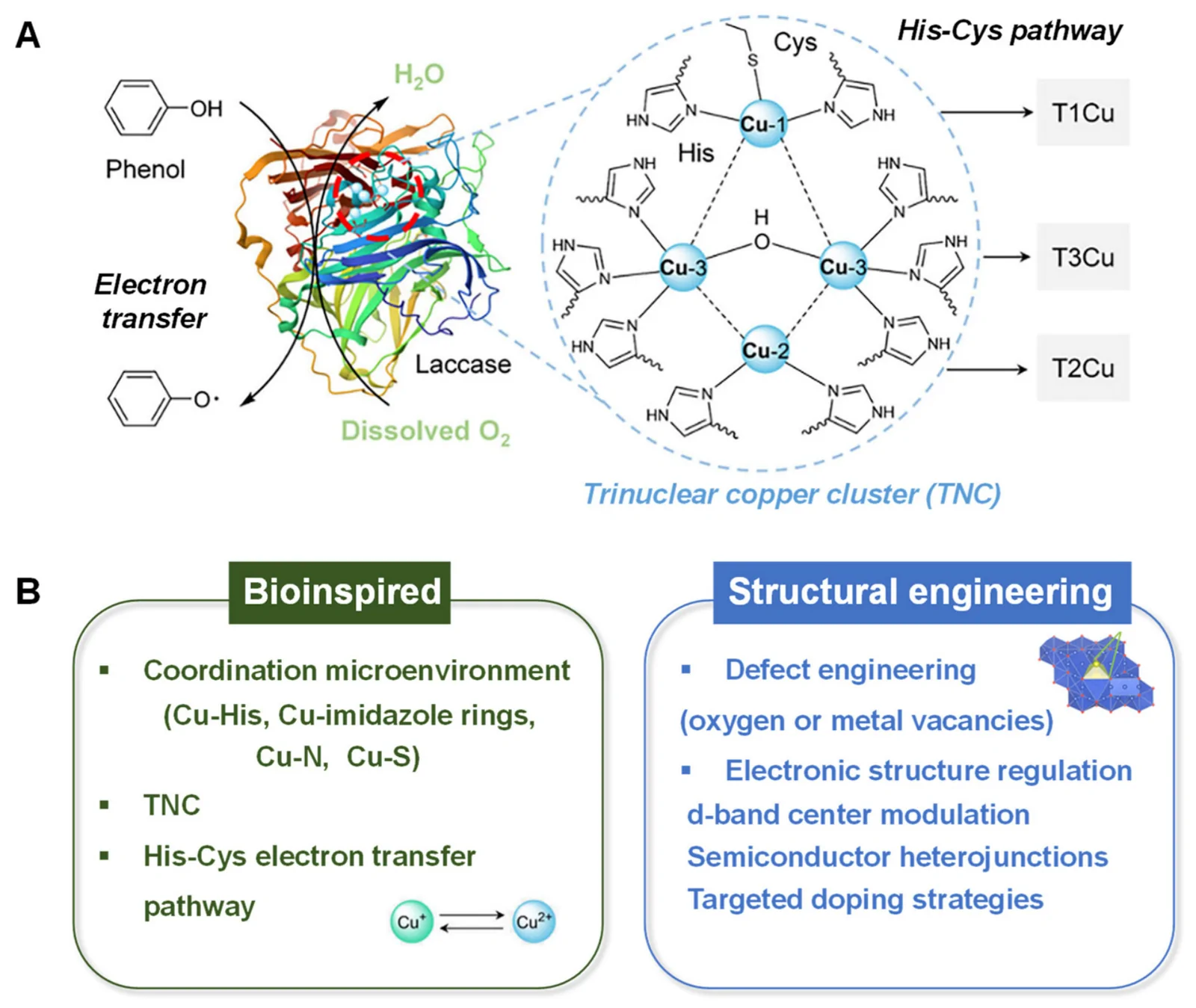
Structural illustration of a typical natural laccase ((**A**), PDB accession number: 1V10) and design strategies of laccase-like nanozymes (**B**).

LLNs represent a class of functional nanomaterials that mimic the catalytic behavior of natural laccase while integrating the advantageous attributes of nanomaterials, such as exceptional stability, cost-effectiveness, scalable production, and facile recyclability. For the development of LLNs, understanding the chemical mechanism of natural laccase is primary and paramount. Natural laccases exhibit remarkable catalytic efficiency, owing to their highly organized multi-copper centers, precise coordination environments, and well-defined electron-transfer pathways (Figure 2A). Structurally, laccase features a tetranuclear copper cluster within its active site, namely one blue type I (T1Cu), one colorless type II (T2Cu), and two type IIIs (T3Cu) [28]. T1Cu is coordinated to histidine (His) and cysteine (Cys), whereas T2Cu and T3Cu form a trinuclear copper cluster (TNC) coordinated to His residues [2]. During catalysis, the substrate is oxidized near T1Cu, and electrons are transferred to the TNC via the Cys–His pathway, where dissolved O_2_ undergoes reduction to form water (H_2_O). Surrounding coordinated amino acid residues also participate in electron transfer and proton transfer, aiding the reduction and cleavage of the O–O bond.

Although LLNs reproduce several key functions of natural laccases, their catalytic structures are not necessarily equivalent. Natural laccases possess well-defined multicopper centers, precisely organized coordination environments, and regulated intraprotein electron-transfer pathways. In contrast, the active sites of many LLNs are structurally heterogeneous and remain incompletely identified. Consequently, relying solely on phenolic-substrate oxidation to assign laccase-like activity may oversimplify the underlying catalytic mechanism. Therefore, LLNs design should focus not only on increasing apparent catalytic activity, but also on establishing experimentally validated relationships among active-site structure, oxygen activation, electron transfer, and substrate oxidation.

Based on the underlying catalytic mechanisms, current design strategies for LLNs can be categorized into two types: Bioinspired and Structural engineering (Figure 2B). The essence of the bioinspired approach lies in mimicking the microenvironment of the natural laccase active center, such as Cu-N coordination, TNC, and His–Cys electron-transfer pathway. The structural engineering strategy primarily employs nano-structural engineering and electronic structural modulation to endow nanozymes with laccase-mimicking catalytic activity. This section systematically discusses the design strategies for LLNs.

#### 2.1.1. Bioinspired Design Strategies

**Figure 3:**
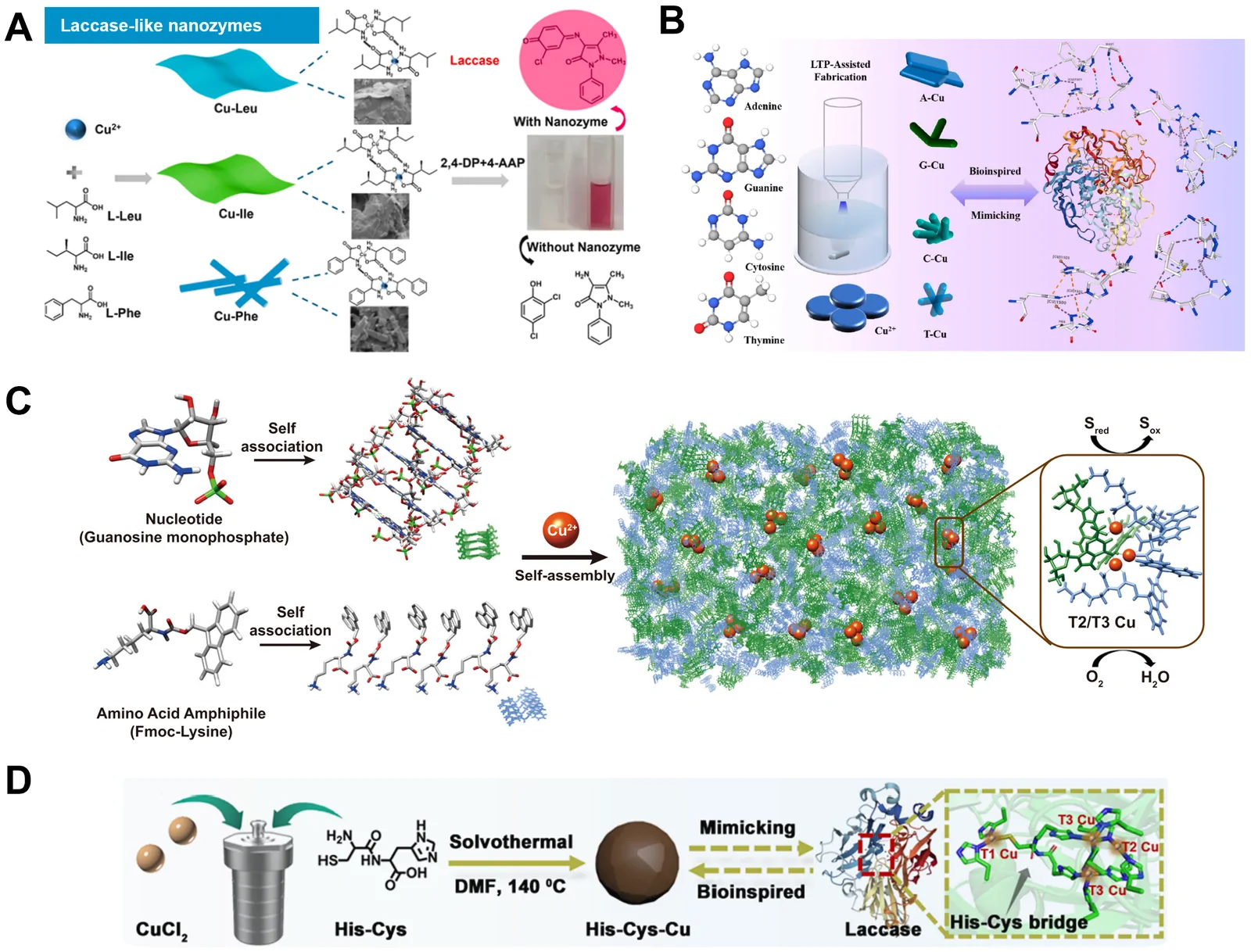
Schematic illustrations of LLNs construction using diverse coordination ligands via bioinspired strategies: (**A**) Amino acids. Adapted with permission from Ref. [33]. Copyright 2025 Elsevier. (**B**) Bases. Adapted with permission from Ref. [13]. Copyright 2025 Elsevier. (**C**) Guanosine monophosphate and modified amino acid. Adapted with permission from Ref. [41]. Copyright 2023 Elsevier. And (**D**) Cys–His dipeptide. Adapted with permission from Ref. [42]. Copyright 2019 Elsevier.

Inspired by the structural and functional characteristics, bioinspired strategies for designing LLNs mainly focus on reconstructing Cu coordination environments, mimicking multicopper catalytic centers, and regulating electron-transfer processes. To mimic the multicopper coordination environment of natural laccase, researchers have explored a diverse array of biomimetic ligands, including natural amino acids (such as His, L-leucine, and L-phenylalanine), unnatural amino acid (such as benzophenone-alanine [29]), peptides [30], and proteins (such as bovine serum albumin [31] and Zein [32]), as coordination ligands for copper ions to construct LLNs. For example, three efficient LLNs were constructed using L-leucine, L-isoleucine, and L-phenylalanine as coordination ligands [33], which self-assembled into the Cu–N coordination environment (as shown in Figure 3A). L-phenylalanine has been reported to be coordinated with Cu^2+^, forming a 2D layered structure that is stacked together through aromatic interactions [34]. Additionally, a series of nucleotides, including adenosine monophosphate [35], guanosine monophosphate [36,37], adenosine diphosphate [38], adenosine triphosphate, nicotinamide adenine dinucleotide [39,40], and pyrimidine derivatives [13], can serve as coordination scaffolds for the construction of LLNs. For example, as illustrated in Figure 3B, the fabrication of four kinds of base-ligand LLNs has been bioinspired, and they have been fabricated via a low-temperature plasma-assisted (LTP-assisted) approach [13].

Moreover, small chemical molecules that contain an imidazole ring, such as imidazole [43], 2-methylimidazole [44], melamine [45,46] and 4,4′-bipyridine [47], have been employed as coordinating ligands for constructing LLNs. Some carboxylic acids, such as L-aspartic acid [48,49], gallic acid [50], vanillic acid [51], and citric acid [52], exhibit excellent metal-coordination capabilities, rendering them promising ligands for designing LLNs. To achieve superior catalytic performance or multifunctionality, dual-ligand strategies have been adopted. As illustrated in Figure 3C, fluorenylmethyloxycarbonyl (Fmoc)-modified amino acid components have been jointly used as complementary ligands to construct LLNs [41]. In other reports, 2-aminoterephthalic acid has been introduced as a secondary ligand alongside His to construct laccase-mimicking materials with fluorescence properties [53,54]. To mimic the Cu–S in T1Cu site, S-containing ligands such as glutathione [55,56], 2-amino-1,3,4-thiadiazole [19], and thioglycolic acid [57], are introduced to regulate the ratio of Cu^+^/Cu^2+^ due to the thiol group. The Cu^+^ is bound to the –SH group via chemical binding, due to the reducibility of –SH, and the Cu^2+^ is bound to the –NH_2_ group via a coordinate bond [55]. The S 2p XPS spectrum confirmed the presence of Cu–S coordination as evidenced by the peak at ~163.3 eV, and further indicated that S-containing ligand is responsible for the high percentage of Cu^+^ [19,42,56]. To mimic the His–Cys electron-transfer pathway, His–Cys dipeptide was used to construct LLNs [42,58]. For example, His–Cys–Cu, which consists of atomic Cu active centers and the His–Cys dipeptide-organized electron-transfer pathway (Figure 3D), exhibits high catalytic activity and robust stability [58]. His–Cys realizes the catalytic oxidation of the substrate by transferring electrons to oxygen and reduces the electrochemical oxidation of 2,4-DP, leading to a decrease in the current.

Overall, bioinspired strategies offer clearer structural links to natural laccases and enable rational regulation of metal coordination and electron-transfer pathways. However, their performance is highly dependent on ligand structure, metal-to-ligand ratio, assembly conditions, and the stability of the resulting coordination environment. Moreover, reproducing individual Cu–N or Cu–S coordination motifs does not necessarily recreate the complete spatial organization and cooperative function of the natural multicopper center. Therefore, improved biomimetic precision should be accompanied by direct active-site characterization and mechanistic validation.

#### 2.1.2. Structural Engineering Design Strategies

Unlike bioinspired strategies that primarily focus on reconstructing the structural and functional characteristics of natural laccase, structural engineering strategies mainly rely on rational architectural design and electronic structure modulation to optimize the catalytic performance of LLNs. By introducing defects, regulating surface electronic states, adjusting band structures, and constructing heterointerfaces, these approaches can create additional active sites, improve substrate adsorption, accelerate electron transfer, and enhance oxygen activation processes. Such material-oriented strategies provide versatile approaches for overcoming the intrinsic catalytic limitations of conventional LLNs and achieving high-performance catalytic systems [59].

**Figure 4:**
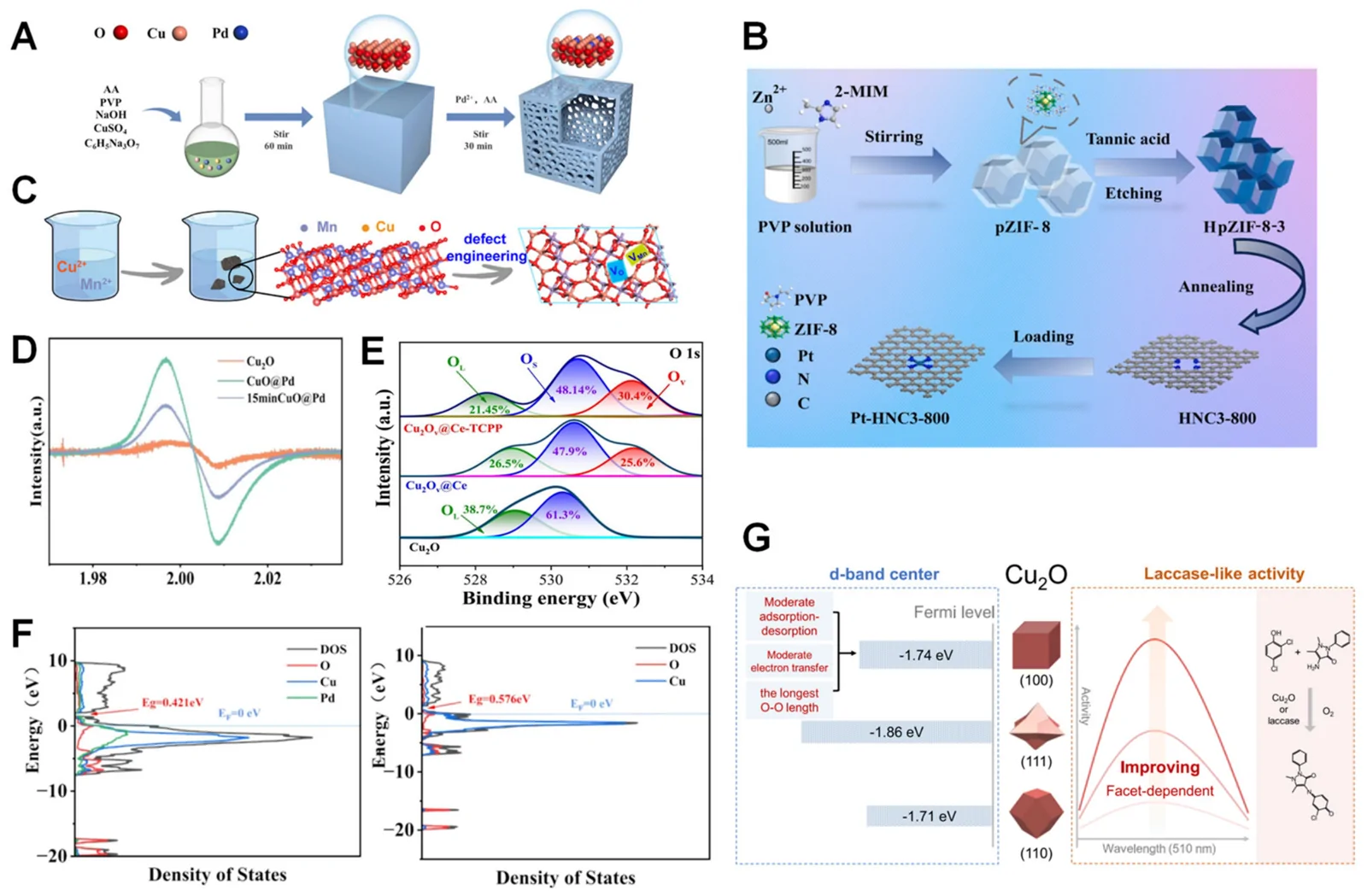
Schematic diagrams of the synthesis of (**A**) CuO@Pd nanocages, (**B**) Pt-HNC3-800 and (**C**) D-CuMnOx. (**A**–**C**) are adapted with permission from [60,61,62], respectively. EPR spectra (**D**) of the synthesized Cu_2_O, CuO@Pd and CuO@Pd-15 min nanocomposites. Adapted with permission from Ref. [60]. Copyright 2025 Elsevier. O 1 s spectrum (**E**) of Cu_2_O, Cu_2_Ov@Ce and Cu_2_Ov@Ce-TCPP. Adapted with permission from Ref. [67]. Copyright 2024 Elsevier. Partial density-of-state (**F**) curves for Cu_2_O and CuO@Pd structures. Adapted with permission from Ref. [60]. Copyright 2025 Elsevier. Schematic diagrams (**G**) of the correlation between d-band center and laccase-like activity of Cu_2_O. Adapted with permission from Ref. [68]. Copyright 2026 Elsevier.

Defect engineering, particularly oxygen vacancy construction, enhances laccase-like activity by modulating coordination environments, electronic density, and surface adsorption, thereby promoting molecular diffusion and electron transfer. Chemical etching and reconstruction strategies are widely employed to generate defect-rich LLNs with improved catalytic performance. For example, hollow porous CuO@Pd nanocages were fabricated through oxygen defect engineering and composition regulation (Figure 4A) [60]. The optimized porous 3D architecture increased the exposure of active sites, while oxygen vacancies facilitated electron transfer and oxygen activation, resulting in enhanced laccase-like catalytic performance. Similarly, a Pt-HNC3-800 nanozyme was constructed by integrating defect-engineered nitrogen-doped carbon frameworks with atomically dispersed Pt–N_4_ coordination sites (Figure 4B) [61]. The integration of hierarchical porous structures, defect engineering, and precisely controlled metal coordination modulated the electronic properties of catalytic centers, thereby enhancing laccase-mimicking activity. Furthermore, a defective Cu-doped Mn-based oxide nanozyme (D-CuMnOx) was developed through simultaneous metal doping and vacancy engineering, where the generation of Mn vacancies and oxygen vacancies introduced abundant Lewis acidic/basic sites and reduced the activation energy for catalytic oxidation (Figure 4C) [62]. Beyond the generation of additional active sites, defect engineering also provides an effective strategy for regulating the electronic structures of catalytic materials. The introduction of oxygen vacancies, metal vacancies, and heteroatom dopants can alter charge distribution, band structures, and metal–oxygen interactions, thereby improving substrate adsorption and oxygen utilization efficiency [21]. The introduction of oxygen vacancies [60,63,64,65], metal vacancies [62,66], or heteroatom dopants [22,25,26,67] can modify the local electronic density and band structure. For example, oxygen defect engineering enables precise modulation of the coordination environments and dynamic equilibria among oxygen vacancies (O_v_), lattice oxygen (O_L_), and adsorbed oxygen (O_S_), thereby accelerating the electron-transfer rate and O_2_ adsorption, lowering reaction energy barriers, and enhancing catalytic activity [60,67]. As presented in Figure 4D, the characteristic symmetric electron paramagnetic resonance (EPR) signal at *g* value of 2.003, which is attributable to unpaired electrons trapped in O_V_, was detected in the CuO@Pd nanocage. The high surface-area porous frameworks of CuO@Pd nanocage enhanced its O_V_ accessibilities. Similarly, upon doping Ce^3+^ and TCPP into Cu_2_O, the O_V_ rate of Cu_2_O@Ce-TCPP increased to 30.4% (Figure 4E), conferring enhanced activity [67].

Electronic structure engineering, guided by d-band center theory, optimizes the adsorption strength of intermediates on catalytic surfaces. Overly high or low d-band centers result in excessive binding or insufficient activation, respectively. Thus, electronic structure engineering aims to optimize the d-band center for enhanced turnover. For example, Pd incorporation into Cu_2_O nanostructures altered the electronic structure and increased the density of states near the Fermi level, thereby improving surface reactivity and electron conductivity (Figure 4F) [60]. Zhang et al. demonstrated that the high laccase-like activity of Cu_2_O nanocubes originated from the exposed (100) facet, whose optimized d-band center (−1.74 eV) facilitated efficient substrate adsorption and oxygen activation (Figure 4G) [68]. This electronic configuration establishes an optimal balance between substrate adsorption and oxygen activation, thereby enabling superior catalytic performance.

Furthermore, heteroatom doping and heterostructure construction represent effective approaches for manipulating electronic structures and promoting interfacial charge transfer [14]. The formation of heterojunctions can induce built-in electric fields, accelerate electron migration, and facilitate oxygen activation at catalytic interfaces. In semiconductor heterostructures, band alignment between different components enables directional charge redistribution, thereby enhancing catalytic oxidation performance. For example, p–n heterostructured nanozymes composed of Co_3_O_4_ and VN were reported to promote electron transfer across the interface, facilitating oxygen adsorption and activation on catalytic surfaces [69]. Similar electronic modulation strategies have also been explored in metal-doped and multicomponent coordination systems, where synergistic interactions between different components improve catalytic activity and stability.

Compared with bioinspired design strategies, structural engineering design strategies provide greater flexibility for regulating defects, electronic structures, and interfacial charge transfer, and may achieve high catalytic activity without reproducing the natural laccase architecture. Nevertheless, the coexistence of multiple defects, metal valence states, and heterogeneous surface sites often complicates the identification of the true catalytic centers. In addition, improved activity observed with model substrates does not necessarily translate into enhanced selectivity or reliability in complex food matrices. The selection between bioinspired and structural engineering strategies should therefore depend on whether mechanistic controllability, catalytic performance, analytical selectivity, or scalable preparation is the primary objective.

### 2.2. Material Types Based on Structural Features

**Figure 5:**
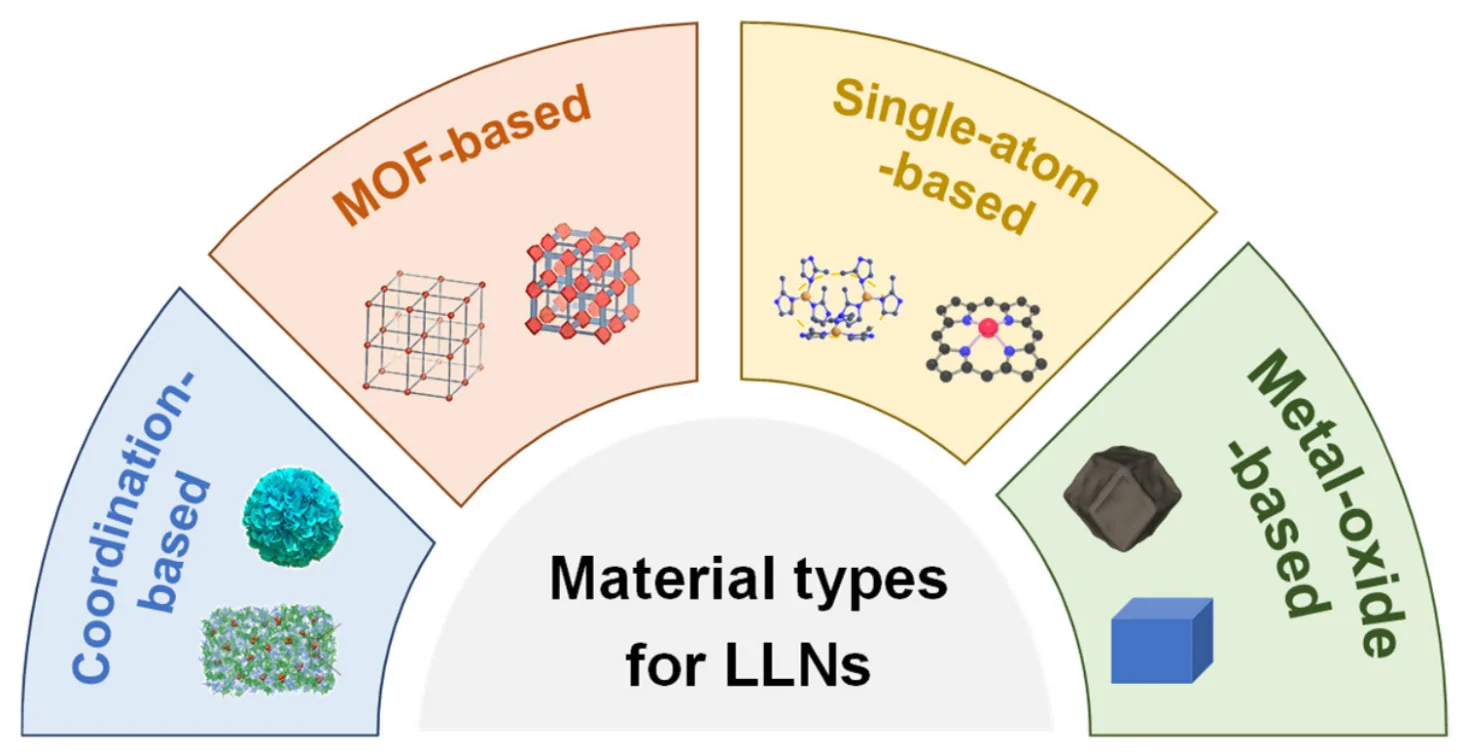
Structural classification of the material types for LLNs.

LLNs represent a rapidly advancing class of functional nanomaterials designed to mimic the catalytic activity of natural laccases while addressing the limitations of natural laccases. Given that the catalytic performance of LLNs is intrinsically dictated by their material composition and atomic structure, a systematic categorization of reported LLNs is essential. Based on their structural features, the reported LLNs fall into four major categories: coordination-based, MOF-based, single-atom-based, and metal-oxide-based LLNs (Figure 5).

Coordination-based LLNs. The well-defined coordination structure in natural laccases provides a fundamental blueprint for the rational design of LLNs. Inspired by this paradigm, a wide array of micromolecular ligands (such as nucleotides, amino acids, peptides, and small chemicals) and macromolecular ligands (such as proteins, DNA, and polydopamine) have been exploited for the construction of LLNs. Typically, micromolecular ligands are coordinated with metal ions via hydrothermal or solvothermal synthetic strategies, whereas macromolecular ligands are assembled through self-assembly processes. For examples, using Cys–His dipeptide as the coordination ligand, a LLN designated as Cu-BH was fabricated via the hydrothermal route at 120 °C for 8 h [53], whereas another system, His–Cys–Cu, was constructed through a solvothermal approach at 140 °C for 5 h [58]. In a distinct strategy, the LLN denoted as Fmoc-K/GMP/Cu^2+^ was assembled via self-assembly of nucleotides and amphiphilic amino acids [41]. Built upon these diverse coordination ligands, LLNs leverage self-assembly to modulate their coordination microenvironment, thereby enhancing their catalytic activity and stability.

MOF-based LLNs. MOFs are a class of crystalline porous materials formed via the self-assembly of metal ions or clusters (nodes) and organic bridging ligands [70,71]. Their exceptional structural diversity, ultrahigh surface area, tunable porosity, and designable chemical environments make them prominent candidates for laccase mimetics [72]. In the MOF-based LLNs, the metal nodes act as intrinsic catalytic sites, while the organic linkers can be selected or functionalized to facilitate substrate adsorption and create laccase-like microenvironments [73]. This strategy not only enhances the stability and reusability of the catalyst, but also allows for systematic optimization of the catalytic activity and selectivity by precisely engineering the framework’s composition and structure at the molecular level [59]. For example, employing 1,3,5-benzene tricarboxylic acid (H_3_BTC) as the ligand, a series of Cu-MOFs exhibiting laccase-like activity have been successfully constructed [74,75,76,77]. Beyond copper, other transition metals, such as Fe, Mn, Ce, Co and V, also exhibit multi-valent features that enable them serve as catalytic centers in LLNs [65,78]. For instance, two multivalent Ce-MOF-based nanozymes, Ce-UiO-66 and Ce-MOF-808, were rationally designed and exhibit superior catalytic performance to natural laccase (from *Trametes versicolor*) [79,80]. Bimetallic MOFs-based LLNs generally outperform monometallic counterparts by offering increased metal active sites and optimized electronic structures through synergistic intermetal interactions. For example, CeCo-MOF exhibits superior laccase-like activity and stability relative to Ce-MOF [81]. Similarly, Cu/Zn-ZIF demonstrates enhanced laccase-mimetic performance, attributed to abundant Cu active sites and the Zn-based MOF backbone that ensures high dispersion of catalytic centers [82]. By incorporating DNA-Cu complexes into a Co-MOF matrix, a core-shell architecture that circumvents conventional pH constraints was constructed [83]. Overall, the catalytic activities of MOF-based LLNs are synergistically governed by both the number and spatial distribution of active sites.

Single-atom-based LLNs. SAzymes feature atomically dispersed metal sites on suitable supports, affording well-defined geometric structures and tunable electronic properties [84]. This atomic dispersion maximizes metal utilization efficiency and exposes abundant catalytic sites, conferring high catalytic efficiency and selectivity [15]. Carbon materials and MOFs are commonly employed as supports to stabilize single atoms via spatial confinement, while optimized precursor design and robust coordination environments further enhance the anchoring strength and electronic stability. In the reported single-atom-based LLNs, isolated metal atoms are typically anchored on nitrogen-doped carbon (N/C) supports derived from carbon materials or MOF precursors, such as graphene oxide [85], ZIF-8 [15,61,86], and ZIF-67 [87]. The formation of geometrically defined M-N_x_ sites (e.g., Cu-N_4_, Fe-N_4_, and Pt-N_4_), which resemble the enzymatic active centers of natural laccase, provides well-defined catalytic centers for laccase-like oxidation reactions. For example, a SAZyme (CuN_4_-G) exhibiting laccase-like activity was deliberately designed and synthesized via thermal treatment of the graphene oxide supporter and copper source at 750 °C in NH_3_ [85]. Strategies including axial ligand configuration, defect engineering, and bimetallic-site construction have been employed to enhance the biomimetic precision of SAzymes by optimizing their electronic structures and reaction pathways. For instance, graphdiyne induces d-π orbital hybridization with copper nanoparticles (Cu NPs) [88]. The Fe-SAzyme with well-defined O_2_–Fe–N_4_ sites exhibits excellent laccase-like catalytic activity [15]. The defect-engineered Pt SAzyme (Pt-HNC_3_-800) with Pt-N_4_ coordination exhibits enhanced catalytic efficiency and stability [61], with density functional theory (DFT) calculations revealing that Pt-N_4_ sites lower the activation barrier for phenolic substrate oxidation via semiquinone radical generation. Beyond copper, other metals, such as Fe, Co, Pt, and Ru, have been used to construct single-atom-based LLNs. For example, Ru SA/GFs exhibit exceptional laccase-like activity due to high substrate affinity and accelerated kinetics [89]. Bimetallic LLNs, such as VCo-N-C [87] and S-FeCo-NC [90], outperform monometallic counterparts through synergistic intermetal interactions, with the latter also displaying additional enzymatic activities.

Metal-oxide-based LLNs. Metal oxide nanomaterials facilitate electron transfer and oxygen activation via inherent properties, rich surface chemistry, and mixed-valence cations, emulating natural laccases. Their straightforward synthesis, robust stability, and composition-tunable activity offer a scalable alternative to biomolecular or coordination-based nanozymes. For example, Liu et al. reported amorphous Co_3_O_4_ with superior laccase-like activity [63], Chen et al. developed N-doped MnO_2_ nanoflowers via a one-step hydrothermal approach [25], and Wu’s research group developed cubic and spherical Ag_2_O nanoparticles [91,92,93], as well as Mn_3_O_4_ [94]. Beyond single-metal oxides, multi-metal oxides have emerged, achieving synergistic catalytic effects. Indeed, multi-metal oxides with multiple catalytic sites show enhanced catalytic activity, attributed to the variable valence states and synergistic interactions among metal components. Based on this, a bimetallic oxide-based LLN, AgCo@CQAS, was developed via a wet-chemical method [66]. The above examples demonstrate that composition regulation, defect engineering and nanostructure design can yield highly efficient and multifunctional LLNs to rival the functionality of their natural counterparts.

These four material classes exhibit distinct advantages and limitations. Coordination-based LLNs are structurally tunable and relatively accessible, but may suffer from limited stability and heterogeneous assembly. MOF-based LLNs provide high surface areas, designable pores, and controllable coordination environments, although mass-transfer limitations and framework instability may occur under certain conditions. Single-atom-based LLNs maximize metal utilization and provide relatively well-defined active sites, but their preparation and active-site characterization are often technically demanding. Metal-oxide-based LLNs generally offer simple synthesis and high stability, whereas their heterogeneous surfaces make precise mechanistic interpretation difficult. No material class is universally superior; material selection should be guided by the target analyte, food matrix, required selectivity, fabrication reproducibility, and intended sensing format.

## 3. Sensing Modes Based on LLNs

**Figure 6:**
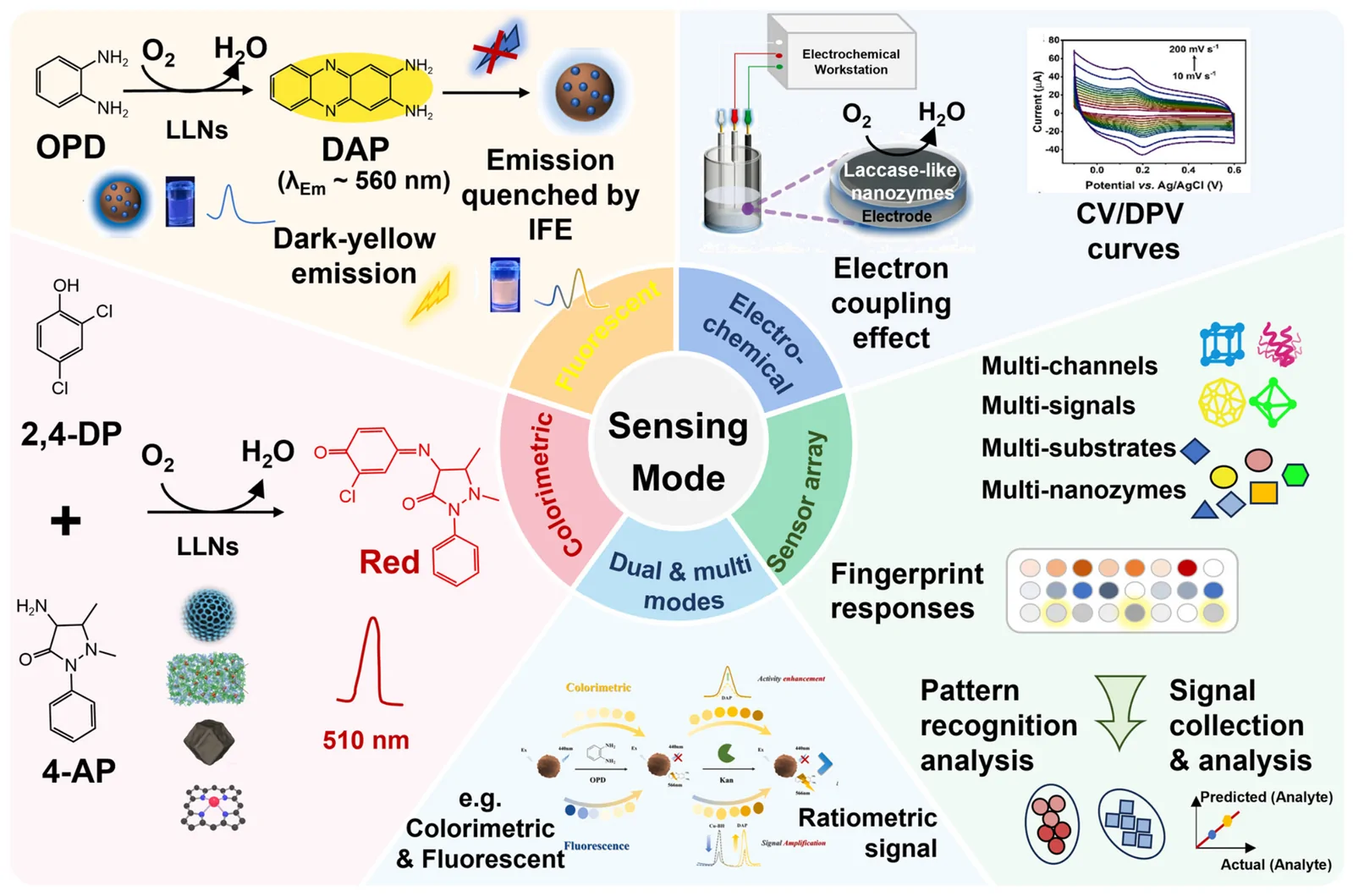
Schematic illustration of sensing modes based on LLNs.

Many LLNs can catalyze the oxidation of target analytes using O_2_ as the terminal electron acceptor, without requiring exogenous H_2_O_2_ as an oxidant. Therefore, LLNs have been widely employed in various sensing platforms. As shown in Figure 6, the sensing modalities based on LLNs can be categorized into colorimetric mode, fluorescent mode, electrochemical mode, sensor array mode, and dual/multi-mode.

### 3.1. Colorimetric-Mode Sensing

Colorimetric sensing, grounded in the measurement of the absorption characteristics of colored substances, is the earliest and most mature mode among LLN detection strategies [95,96]. Colorimetric sensing enabled by LLNs commonly employs a well-established chromogenic system based on the oxidative coupling of 2,4-dichlorophenol (2,4-DP) and 4-aminoantipyrine (4-AP) (Figure 6). In the assay, LLNs catalyze the oxidation of 2,4-DP to intermediates, which couple with 4-AP to form a red quinone-imine dye with a characteristic absorption peak at 510 nm [42]. Owing to its simplicity, rapid response, low cost, and compatibility with portable and high-throughput analysis, LLN-based colorimetric sensing has been extensively applied for the detection of various targets [97]. Moreover, the measurable chromogenic reaction enables the evaluation of catalytic properties, where kinetic parameters such as the Michaelis constant (*K*_m_) and maximum reaction velocity (*V*_max_) can be obtained through Lineweaver–Burk analysis [98], providing quantitative insights into catalytic efficiency.

### 3.2. Fluorescence-Mode Sensing

Compared with colorimetric detection, fluorescence detection typically exhibits higher sensitivity and accuracy in complex biological and food matrices, making it more suitable for complex food systems [99]. Since coexisting components such as natural compounds and food additives may interfere with the detection process, the development of rapid, accurate, and highly sensitive analytical methods is of great significance [100]. Accordingly, LLN-based sensing strategies involving catalysis and fluorescence signal conversion have gradually garnered attention, providing an effective platform for the highly sensitive detection of complex food samples. In the LLN-based sensing, the incorporation of fluorescent materials/molecules, intrinsic ligand fluorescence, or the fluorescent properties of enzymatic reaction products enables the establishment of a fluorometric detection approach. For instance, 2-aminoterephthalic acid (BDC-NH_2_), which imparts the blue fluorescent functionality (emission at ~425 nm), has been used either as the sole coordination ligand [101] or alongside another ligand [53,54,102] with copper ions, to construct fluorescent LLNs. Fluorescence sensing based on LLNs commonly relies on the oxidation of the non-fluorescent substrate o-phenylenediamine (OPD) to 2,3-diaminophenazine (DAP), which exhibits fluorescence emission at approximately 560 nm (Figure 6). The generated DAP could quench the nanozymes’ fluorescence by the fluorescence internal filtration effect (IFE) [53,54]. The concentration of analytes correlates with the ratiometric fluorescence signal (e.g., F_560_/F_425_) [103]. More recently, this strategy provides complementary contrasting data to a colorimetric signal, thereby establishing a self-verifying dual-mode sensing platform to enhance analytical accuracy and reliability. Moreover, the LLN-based ratiometric fluorescence sensing attracts increasing attention, due to the merits of LLNs, like providing an intrinsic self-referencing way to markedly improve resistance to environmental and instrumental fluctuations [53,54]. In addition, functionalized fluorescent nanomaterials such as carbon quantum dots [104], with their excellent luminescent properties and molecular recognition capabilities, offer new strategies for expanding LLN-based fluorescent sensing systems and developing highly sensitive, multifunctional food-safety detection platforms.

### 3.3. Electrochemical-Mode Sensing

Electrochemical-mode sensing has emerged as a powerful and rapidly advancing analytical technique, characterized by high sensitivity, robust reliability and cost-effectiveness [105]. Electrochemical sensing employing LLNs operates on the catalytic oxidation of electron-donating analytes, like thiram [77], guaiacol [85], quercetin [106], or other phenolic compounds, converting their chemical oxidation into quantifiable electrical signals. LLNs can be directly anchored on the surface of a glassy carbon electrode (Figure 6). Typically, electrochemical sensors are modified with diverse LLNs, to enable the precise detection of target analytes. For example, a CuN_4_-G-modified LLN electrochemical sensor enabled picomolar-level guaiacol detection, with DFT confirming that bonding-electron coupling enhanced electron transfer, conductivity, and catalytic performance [85]. A bioinspired electroactive LLN prepared from Fe_3_O_4_/Cu-MOF enabled rapid ratiometric electrochemical detection of thiram via analyte-induced catalytic inhibition and conductivity enhancement, permitting direct detection on the electrode, strong thiram–Cu^2+^ affinity for rapid and selective recognition, and binding-induced dual-signal output [77]. These LLNs facilitate laccase-like oxidation of substrates, during which electrons are sequentially abstracted from the substrates and transferred through the catalytic centers, ultimately reducing dissolved O_2_ to water and generating measurable electrochemical signals proportional to the analyte concentration. This process is usually reflected in measurable electrochemical responses obtained using techniques such as cyclic voltammetry (CV), differential pulse voltammetry (DPV), electrical impedance spectroscopy (ESI), and square wave voltammetry (SWV). These studies demonstrate the potential of LLNs as catalytic elements for electrochemical analysis in complex food matrices [77,85,106,107,108,109].

### 3.4. Sensor Array Mode

The sensor array operates on the principle of differential cross-responsive interactions between multiple sensing units and target analytes [110,111,112], which, when coupled with pattern recognition algorithms, generate unique multidimensional fingerprints that enable reliable qualitative and semi-quantitative analysis, even in complex matrix environments (Figure 6). In recent years, nanozyme-based sensor arrays have become a key intelligent detection technology in various fields, including food quality control and safety inspection [113]. Sensor array platforms based on the multi-enzymatic activities of a single LLN [50,63,101] or the cross-response of multiple LLNs [38,40,114] have been developed for selective recognition of analytes. For example, a three-channel colorimetric sensor array was constructed, using the reaction kinetics of a LLN (GA-Cu) as the sensing unit, for the detection of six phenolic acids in juice, beer and wine samples [50]. A four-channel sensor array was established using four LLNs (GMP-Cu, BPY-Cu, MIZ-Cu, and ASP-Cu), enabling highly selective identification and discrimination of organophosphorus pesticides [114]. To further enhance sensor sensitivity and detection performance, statistical methods and machine learning algorithms are integrated with sensor arrays. By analyzing vast amounts of image/absorbance data, machine learning algorithms enhance accuracy and minimize errors in signal recognition. Various machine learning classification models, including Artificial Neural Network (ANN) [51,76], Hierarchical Cluster Analysis (HCA) [60,63,81], K-Nearest Neighbors (KNN) [115,116], Linear Discriminant Analysis (LDA) [38,50,81,101,114,117,118], Random Forest (RF) [39,40,75,119,120], Principle Component Analysis (PCA) [25,101], Support Vector Machine (SVM) [46,115,116], You Only Look Once version 8 (YOLOv8) [33,121], and other neural network-based approaches [52,75,115,116,120] have proven effective for multiple analyte detection in food samples.

### 3.5. Dual-/Multi-Mode

Compared to the single detection mode, the integration of dual or multiple modes for the development of multi-readout analytical strategies offers substantial advantages, manifesting as enhanced specificity, reliability, applicability, and low detection limits [122,123,124]. Particularly noteworthy, dual-mode and multi-mode sensing improve the applicability of analytical methods for the detection of trace analytes within complex food matrices [121,125,126]. For example, a sensitive dual-mode (colorimetric and fluorescent) sensing system using a LLN (Cu-BH) was established for the detection of kanamycin in milk and honey [53]. This system operates on the principle that kanamycin promotes the catalytic activity of Cu-BH, resulting in a gradual increase in absorbance at 426 nm and an enhanced fluorescence signal at 566 nm. The dual-mode assay based on the ratiometric fluorescence strategy enables cross-referencing of the two modes, establishing built-in corrections for environmental influences and achieving a higher analytical accuracy [127]. Furthermore, nanozyme catalysis can be integrated with surface-enhanced Raman scattering (SERS) to develop highly sensitive sensing platforms, where nanozyme-catalyzed reactions can modulate the intensity of characteristic Raman signals for analyte detection [128]. SERS provides molecular fingerprint information for qualitative identification, while quantitative analysis can be achieved through signal calibration or complementary analytical strategies. Such catalytic–SERS hybrid systems offer promising opportunities for sensitive and selective food-safety sensing [10].

From an analytical perspective, each sensing mode presents specific trade-offs. Colorimetric assays are simple, compatible with portable readout, and require minimal instrumentation, rendering them particularly suitable for large-scale on-site analysis in the food industry. Moreover, integration with smartphones and smartphone-based colorimetric analysis software (ImageJ 1.x) further facilitates their widespread application, especially in developing regions. Fluorescence-based methods generally provide higher sensitivity, although signal stability and background fluorescence remain concerns in complex food samples. Electrochemical platforms offer sensitive quantitative measurements, but require electrodes and instrumentation with reliable inter-batch reproducibility. Sensor arrays enable discrimination of structurally related analytes, but depend strongly on model training, dataset quality, and external validation. Dual-/multi-mode sensing can improve analytical reliability through cross-validation, although the increased system complexity may limit standardization and practical deployment.

## 4. LLNs for Sensing Application in Food Quality and Safety

**Figure 7:**
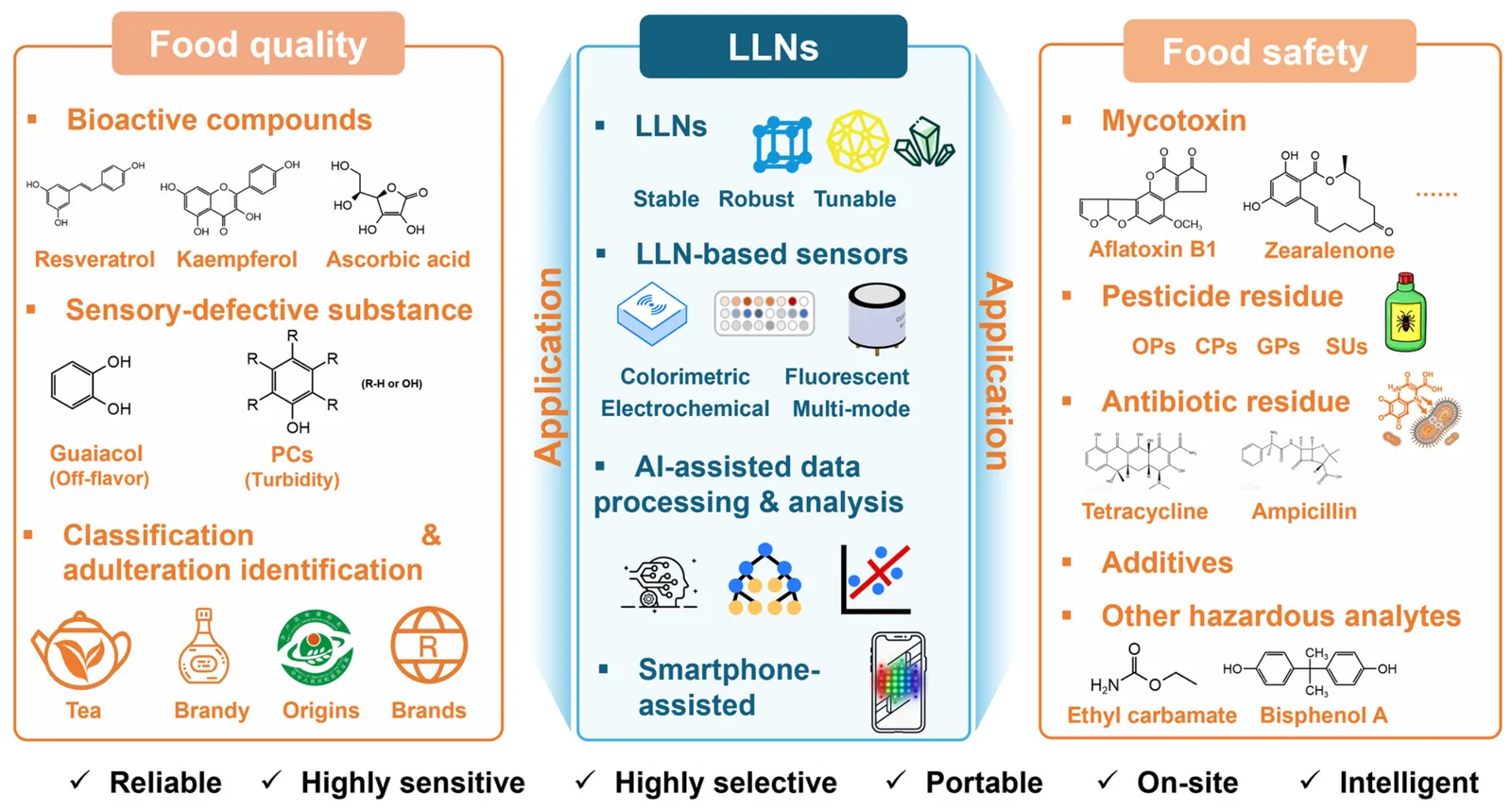
Applications of LLNs-based sensing technologies in food quality and safety monitoring.

Food nutrients and bioactive components, such as phenolics and flavonoids, are pivotal determinants of food quality, contributing to desirable organoleptic properties [129,130] and nutritional value [131,132]. Meantime, adulteration driven by economic gain degrades these very attributes, undermining food authenticity and disrupting the stability of the market ecosystem [133,134,135]. In addition, food safety is critically compromised by contamination (e.g., from mycotoxins, pesticide/antibiotic residues, and packaging migrants) across the production chain, and the overuse of additives [136]. This pressing situation demands a critical re-evaluation and continuous advancement in food analysis methodologies. Conventional techniques like HPLC, GC–MS, and PCR are laboratory-dependent, costly, and operationally complex, restricting their widespread application in developing regions [137,138]. In this context, LLNs, characterized by high sensitivity and specificity, tailorable synthesis, operational simplicity, and eco-friendliness, hold promise for overcoming the current dilemma in analytical technology. Integrating with sensor technologies, artificial intelligence algorithms [88,139,140], and smartphone devices facilitate the reliable, sensitive, intelligent, on-site and portable detection, positioning LLN-based methods as next-generation tools for food quality and safety assurance. Currently, LLNs have attracted considerable research attention and exhibited extensive practical applications in food industry. This section reviews their validated applications in the food industry (Figure 7).

### 4.1. Food Quality

Accurate assessment of food nutrients and chemical components is fundamental to ensuring food quality [141,142]. LLNs, with their superior catalytic tunability, have enabled the development of sensitive, rapid, and portable detection platforms. Table 1 summarizes their reported applications in monitoring nutrition, sensory quality, and authenticity.

#### 4.1.1. Detection of Bioactive Compounds

**Figure 8:**
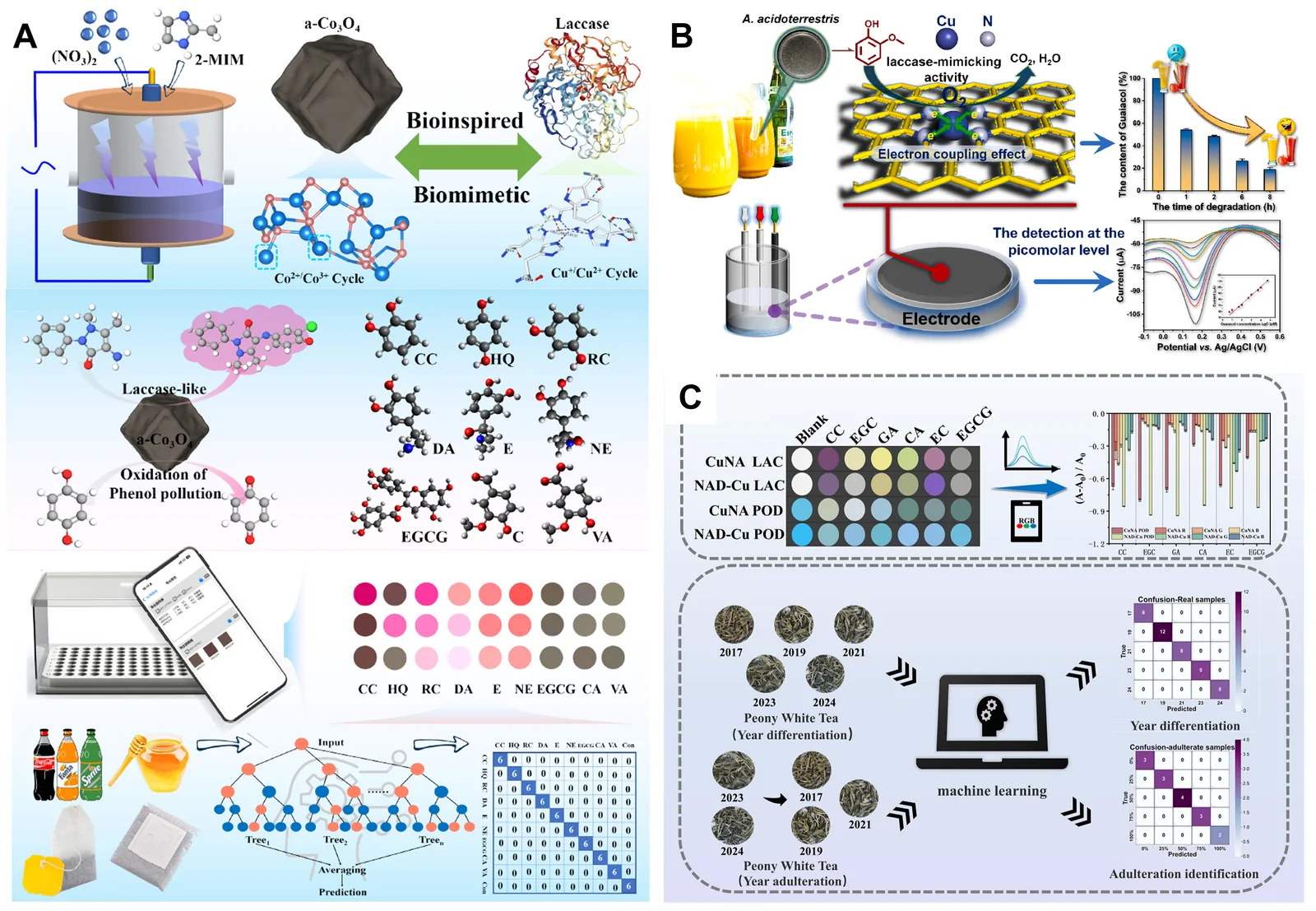
Applications of LLN-based methods in food quality. (**A**) Illustration of a-Co_3_O_4_ application in intelligent identification of PCs [63]. (**A**) Adapted with permission from Ref. [63]. Copyright 2025 Elsevier. (**B**) Schematic of electrochemical sensor based on a monatomic copper LLN for guaiacol detection in food [85]. (**B**) Adapted with permission from Ref. [85]. Copyright 2023 Elsevier. (**C**) Schematic diagram of colorimetric sensor array for identifying six types of tea polyphenols, year differentiation, and adulterated samples [39]. (**C**) Adapted with permission from Ref. [39]. Copyright 2025 Elsevier.

Food matrices constitute rich sources of diverse bioactive constituents, including phenolic compounds, flavonoids, chlorogenic acid, resveratrol, quercetin, and ascorbic acid, etc. Phenolic antioxidants are the most important bioactive compounds in plant-based foods [148,149]. PCs not only shape the taste and quality of various foods, but also confer significant benefits to human health, underscoring the need for effective detection methods [150]. By virtue of the differential laccase-like activity of N-doped MnO_2_ nanoflower towards different PCs, a colorimetric sensor array was constructed [25]. Benefiting from the distinctive fingerprint signals, this method effectively identified and differentiated six PCs in complex food matrices, including coffee, tea, onions and guava. Similarly, based on the differential laccase catalytic efficiencies of the GA-Cu nanozyme towards six PCs, a three-channel colorimetric sensor array using reaction kinetics achieved high-throughput, wide-range detection of PCs, and successfully discriminated six PCs in juice, beer, and wine [50]. By integrating the ANN algorithm, this research group developed a six-channel sensor array using a VA-Cu nanozyme, achieving 100% accuracy in identifying and predicting PCs in blind samples (black tea, honey, and grape juice), thereby enabling reliable profiling in complex food matrices [51]. In a subsequent study, they constructed a bifunctional Cu-BTC nanozyme with laccase/peroxidase-like activities [76]. Inhibition by various PCs enabled a dual-mode colorimetric/photothermal sensor array. Moreover, by combing ANN algorithms with sensor arrays, this platform achieved intelligent recognition of ten PCs in black tea, coffee, and wine. More recently, a colorimetric sensor array employing a-Co_3_O_4_ was developed for the accurate identification of various PCs in food [63]. Taking advantage of the catalytic versatility, stability, and pH-dependent substrate affinity of a-Co_3_O_4_, this method sensitively discriminated nine PCs (Figure 8A). Furthermore, the integration of machine learning with smartphone-based imaging analysis enabled rapid and intelligent identification of PCs in beverage and honey samples with low LODs and nearly 100% recovery rates. Collectively, these studies demonstrate that LLNs-based sensing technology holds considerable promise for identifying various PCs in complex food matrices.

Given the critical role of tannins in shaping the flavor complexity, aging potential, and health-related properties of wine and tea beverages, their detection is essential for quality assurance and regulatory compliance in the beverage industry [151]. A sensitive colorimetric sensor using a dual-functional single-atom Fe nanozyme (Fe@CN-20) with laccase/oxidase-like activities was constructed for tannin quantification in brandy [24]. Utilizing the competitive inhibition between 3,3′,5,5′-tetramethylbenzidine (TMB) and tannins, this method enabled precise tannin quantification in five brandy products, showing high selectivity and reliability. To further extend the detection range for tannins and simplify actual food-sample pretreatment, a colorimetric sensing platform using a bimetallic LLN, VCo-N-C, was developed, based on the shift from the TMB discoloration mechanism to the 4-AP color enhancement principle [87]. Combined with the specific chromogenic agent 4-AP, this platform expanded the detection range to 0.1–3000 mg/L, enabling direct (without pretreatment), rapid (within 10 min), and accurate (comparable results with standard methods) detection of high-concentration tannic acid in brandy, liquor, and tea beverages.

Inspired by natural laccase, four Cu-MOF-based LLNs (A-Cu, G-Cu, C-Cu and T-Cu) were constructed, and their differential reactivity toward bioactive substances was exploited to develop a four-channel colorimetric sensory array for identification of five typical natural bioactive compounds (such as resveratrol, rutin and vanillin) [13]. With the assistance of a smartphone, this platform enabled high-throughput, high-sensitivity, and smart sensing for bioactive compounds in food. Flavonoids are a class of active compounds widely found in foods, with antioxidant, anti-inflammatory, and anti-tumor activities [152,153,154]. A biomimetic electrochemical biosensor was constructed for the sensitive detection of quercetin by leveraging the laccase-like activity of N/Ni single-atom nanozyme [106]. The electrochemical sensor showed good selectivity, reproducibility, repeatability and stability, suggesting remarkable potential for practical applications. A novel CA-Cu nanozyme with dual activities (laccase/peroxidase) was synthesized [52]. Given that flavonoids inhibited these activities time-dependently, a 4-channel nanozyme sensor array was developed for flavonoid identification. Integration with various machine learning algorithms enabled accurate discrimination and prediction of five flavonoids in multiple complex healthy tea, showing strong potential for flavonoid identification in real samples.

Ascorbic acid (vitamin C, AA) plays a very important role in maintaining the normal metabolism of the human body. Leveraging its intrinsic laccase-mimicking activity, CuNi/CoMoO_4_ facilitated the construction of a colorimetric assay for ascorbic acid quantification [16]. The detection mechanism relied on the intense absorbance of the phenol/4-AP/CuNi/CoMoO_4_ system at 510 nm, which was quenched upon introducing ascorbic acid, thereby validating the sensing principle. The proposed platform exhibited exceptional analytical performance, including a superior detection limit and linear dynamic range, alongside high selectivity. Based on the “activation–inhibition” dual regulation mechanism of CeO_2_, a new colorimetric sensing platform for AA detection was developed, enabling high sensitivity, selectivity, and robustness of AA detection in complex food matrices [22]. These findings underscore the significant potential of LLNs for antioxidant capacity assessment within food quality control.

#### 4.1.2. Sensory-Defective Substances

In apple juice and orange juice, high guaiacol levels (>2 PPB) cause off-flavors, and overconsumption risks hepatorenal toxicity. To address this concern, an electrochemical sensor modified with a LLN, CuN_4_-G, was developed [85]. As depicted in Figure 8B, guaiacol oxidation yielded characteristic CV and DPV peaks. The CuN_4_-G-based electrochemical sensor detected guaiacol from 5 to 50,000 pM with a 1.2 pM limit. and was applied to orange juice contaminated with *Alicyclobacillus acidoterrestris* during storage. It displayed high selectivity and excellent stability, underscoring its promise for food quality assessment in complex food matrices.

Residual phenolic compounds in fruit juices can cause turbidity and affect their sensory quality. Therefore, it is necessary to carry out clarification treatment during juice processing. In recent years, laccases and LLNs have been extensively employed for the clarification of fruit juices [155]. The laccase-like activity of AMP-Cu nanozyme outperformed natural laccase from *Aspergillus* sp. in the reduction of phenolic content, highlighting its potential as an efficient alternative for juice clarification [35]. The CYP-Mnzyme enabled the efficient degradation of catechol derivatives in five fruit juices, directly contributing to significant turbidity reduction, offering practical utility in juice processing [144].

#### 4.1.3. Classification and Predication of Food Freshness

During storage, food produces volatile spoilage markers, due to microbial metabolism and oxidative reactions [156]. Therefore, the development of rapid, sensitive quality-monitoring methods is of great significance for food safety assessment. In recent years, nanozyme-based sensing platforms have further advanced the rapid detection of meat freshness [112,157]. For example, MnZn bimetallic cold-adapted nanozymes prepared using the dielectric-barrier discharge plasma method can be used for in situ monitoring of volatile sulfur compounds and for assessing meat freshness [147]. Furthermore, integrating sensing technologies with intelligent packaging strategies may provide a promising approach for real-time monitoring and active preservation of meat quality, through antimicrobial and antioxidant functions [158].

#### 4.1.4. Tea Classification and Adulteration/Authenticity Identification via Flavor Component Sensing

Sensor arrays have been widely employed for the identification of complex flavor components or markers [20,129,159], offering a novel approach to food grading [160,161] and adulteration identification [133,134]. For instance, by exploiting the differential responses of LLNs, the modulating effects of metal ions on their catalytic activity, a 3-channel colorimetric array was developed, enabling the accurate identification of blind tea samples from different origins [38]. Jin et al. constructed a colorimetric sensor array using two LLNs (CuNA and NAD-Cu) to catalyze distinct color reactions between tea PCs and 4-AP (Figure 8C) [39]. Fingerprint responses from six key tea PCs were analyzed via a RF algorithm, achieving 100% accurate discrimination. The method perfectly identified white peony tea adulteration across five levels (0%, 25%, 50%, 75%, and 100%), offering an efficient, stable and convenient adulteration identification method.

### 4.2. Food Safety

The rapid identification of contaminants and harmful components in food is of great importance for ensuring food safety [162]. In recent years, thanks to their excellent catalytic performance and tunable active structures, LLNs have gradually emerged as key materials for developing highly sensitive, rapid, and portable food-safety testing platforms. Table 2, Table 3, Table 4, Table 5 and Table 6 summarize the reported applications of LLNs in monitoring mycotoxin, pesticide residues, antibiotic residues, food additives and other hazardous analytes.

#### 4.2.1. Mycotoxin Detection and Mitigation

To ensure food safety and human health, it is necessary to establish a simple, rapid and sensitive method for mycotoxin detection [163,164]. Fumonisin B1 (FB_1_), deoxynivalenol, zearalenone (ZEN), and aflatoxin B_1_ (AFB_1_), as listed in Table 2, are among the predominant mycotoxins commonly contaminating foodstuffs [165,166,167,168,169,170]. Given that FB_1_ could specifically inhibit the laccase-mimicking activity of Ag_3_PO_4_ nanoparticles, a colorimetric sensor was established for FB_1_ detection [23]. The developed sensor achieved satisfactory sensitivity (LOD: 1.73 μg/L) and accuracy (recovery rates of 87.8–104.5% in corn and wheat samples). Leveraging the magnitude of deoxynivalenol-induced enhancement of the laccase-like activity of FeCu nanozymes, the FeCu nanozyme platform enabled the detection of deoxynivalenol in wheat, bran, and corn, thereby offering a rapid screening tool for safety assessment [171]. The specific interaction between deoxynivalenol and FeCu allows the nanozyme to function as a targeted probe for precise deoxynivalenol quantification, effectively mitigating interference from other mycotoxins. By virtue of the integrated laccase-, phosphatase-, and catalase-like activities of CuNA@Arg-Ce, a self-cascade one-step colorimetric method was established to detect ZEN [65]. This selective interruption of the cascade reaction enables a “signal-off” colorimetric detection mode, achieving a LOD for ZEN (0.157 μg/L) and desirable recovery rates (90.8–107.1%) in multiple grain samples. Additionally, a smartphone-based colorimetric (red, green, blue, RGB) analysis enables rapid, reliable, on-site detection of ZEN in corn samples.

**Table 2:** Applications of laccase-like nanozymes in mycotoxin detection and mitigation in various food matrices.

Mycotoxins	Food Sample	LLNs	Detection Modes	Performances	Refs.
Fumonisin B1	Corn and wheat	Ag_3_PO_4_ NPs	Colorimetric	LOD: 1.73 μg/L, recovery rate: 87.8–104.5%	[23]
Deoxynivalenol	Wheat, wheat bran, corn	FeCu	Colorimetric	LOD: 0.59 μM,	[171]
Zearalenone	Maize, wheat, coix seed, soybean, oats	CuNA@Arg-Ce	Colorimetric	LOD: 0.157 μg/L, recovery rate: 90.8–107.1%	[65]
Aflatoxin B1	Peanut milk, corn juice, crushed corn, white vinegar	Fe/NC SAzyme	/	Removal rate: 96.95% within 1 h	[15]
	Peanut oil	CuTA	/	Removal rate: 78% within 9 h	[172]
		amphiphilic enzyme mimics-based hybrid nanoflower	/	Removal rate: 98% within 3.5 h	[30]

Mold contamination and the toxins it produces are major risk factors for food safety [173]. In addition to suppressing toxin-producing fungi, it is crucial to develop highly effective strategies for detecting and neutralizing mycotoxins [174]. Among various mitigation strategies, LLNs show great potential for the catalytic transformation of mycotoxins. AFB_1_ can be catalyzed into aflatoxin Q1 (AFQ_1_) by Fe-SAzyme, which has been reported to exhibit lower toxicity than AFB_1_ [15]. Fe-SAzyme (25.4 Å) had a larger pore size than the AFB_1_ (8.7 Å × 10.8 Å), favoring its excellent adsorption capacity for AFB_1_. Therefore, Fe-SAzyme showed superior adsorption and removal ability for AFB_1_ in peanut milk, corn juice, crushed corn, and white vinegar. The laccase-mimicking CuTA nanozyme demonstrated high ability to oxidize AFB_1_, achieving a degradation rate of 78% within 9 h [172]. Due to the high surface area, enzyme confinement effect and the amphipathic character, the EmNF-P showed exceptional degradation performance for AFB_1_ in peanut oil without losing nutrients and flavor components [30]. Currently, the laccase-like catalytic mechanism for AFB_1_ degradation have been proposed based on DFT calculations, molecular dynamics simulations, and UPLC-MS results [15,30]. Three degradation pathways have been proposed [172]: (1) cleavage of the lactone ring within the coumarin moiety; (2) C3-hydroxylation to yield AFQ_1_; and (3) epoxidation of the terminal furan ring olefinic bonds, followed by epoxide elimination and demethylation of the benzene ring side-chain methoxy group.

#### 4.2.2. Pesticide Residues

Given the potential health risks posed by pesticide residues in food commodities, the establishment of simple, rapid, and sensitive analytical methods is imperative for effective food safety monitoring and risk assessment [175,176,177]. As shown in Table 3, significant efforts have been made to develop LLN-based methods for pesticide detection.

**Table 3:** Applications of LLNs toward pesticide residue detection in various food matrices.

Pesticides	Food Sample	LLNs	Detection Modes	Performances	Refs.
Thiram	Apple juice and pear juice	GSH-Cu	Colorimetric	LOD: 2 ng/mL, recovery rate: 87.1–106.1%	[56]
	Pear, apple, broccoli, and cucumber	Fe_3_O_4_/Cu-MOF	Electrochemical	LOD: 0.15 nM, recovery rate: 98.4–105.4%	[77]
	Mango and cabbage	Cu@COP	Colorimetric	LOD: 2.46, recovery rate: 91.6–106.5%	[12]
Glyphosate pesticide	Apple, cabbage, and cucumber	Cu-BDC	Colorimetric	Recovery rate: 90.34–96.13%	[178]
Multiple pesticides	Apples, pears, nectarines, celery, tomatoes, and cabbage	GMP-Cu BPY-Cu MIZ-Cu ASP-Cu	Colorimetric	Discriminant accuracy: 100%	[114]
	Cabbages, tomatoes, grapes, and apples	Mel-Cu	Colorimetric	Discriminant accuracy: 100%	[46]
	Vegetables and fruits	DNA-Cu@MOFs	Colorimetric	LOD: 0.75 ng/mL, recovery rate: 97.2–107.9%	[83]
	Broccoli, cabbages, and tomatoes	ASP-Cu	Colorimetric	Discriminant accuracy: 100%	[48]
	Six kinds of fruits and vegetables	Cu-BDC-NH_2_	Dual-mode (colorimetric & fluorescence)	Discriminant accuracy: 100%	[101]
	Soybeans, mung beans, corns, and glutinous rice	ASP-Cu BPY-Cu GMP-Cu	Colorimetric	Discriminant accuracy: 100%	[117]
	Tomatoes and leeks	NAD-Cu ATP-Ce	Colorimetric	Discriminant accuracy: 99.2%	[120]
	Spinach	Cu-carboxylate nanozymes	Colorimetric	Discriminant accuracy: 100%	[119]
	Tomatoes, grapes, and cherries	Cu-amino acid nanozymes	Colorimetric	LOD: 0.0012–0.0080 μM	[33]
	Corn, oat, and jujube	Cu/Ce-MOF Laccase/POD/OXD	Colorimetric	Discriminant accuracy: 100%	[179]
Acetamiprid	Tomatoes, cabbages, and apples	Spherical Ag_2_O NPs	Colorimetric	LOD: 8.69 μg/L, recovery rate: 83.1–94.1%,	[94]
Pyrethroid pesticides	Wheat, sorghum, and corn	AgCo@CQAS	Colorimetric	LOD: 0.11 μg/L, recovery rate: 89.8–95.3%	[66]
Malathion	Apples, orange juice, and cabbage	Ag_2_O NPs	Colorimetric	LOD: 1.95 μg/L	[92]
Organophosphorus pesticides	Apple juice, milk, and tea	His–Cys–Cu	Colorimetric	Recovery rate: 81.4–109.5%	[58]
	Fruits, vegetables, brown rice, cottonseed oil, and tea	Ce-CDs/MnO_2_	Colorimetric	Recovery rate: 84.7–99.8%	[26]
Pyrethroid pesticides	Tea, tobacco, and cabbage	Cu-ATP@[Ru(bpy)_3_]^2+^	Dual-mode (colorimetric & fluorescence)	Recovery rate: 96.0–100.0%	[121]
N-(phosphonomethyl) glycine pesticides	Apple and cabbage	Mn-uNF/Si	Colorimetric	LOD: 0.61 μM,	[64]
Dimethoate	Cowpea, cabbage, and pepper	AgCit	Colorimetric	LOD: 1.0 μg/L	[180]
Thiophanate methyl	Apples and tomatoes	Cu-BH	Fluorescence	LOD: 0.03 μM	[54]

**Figure 9:**
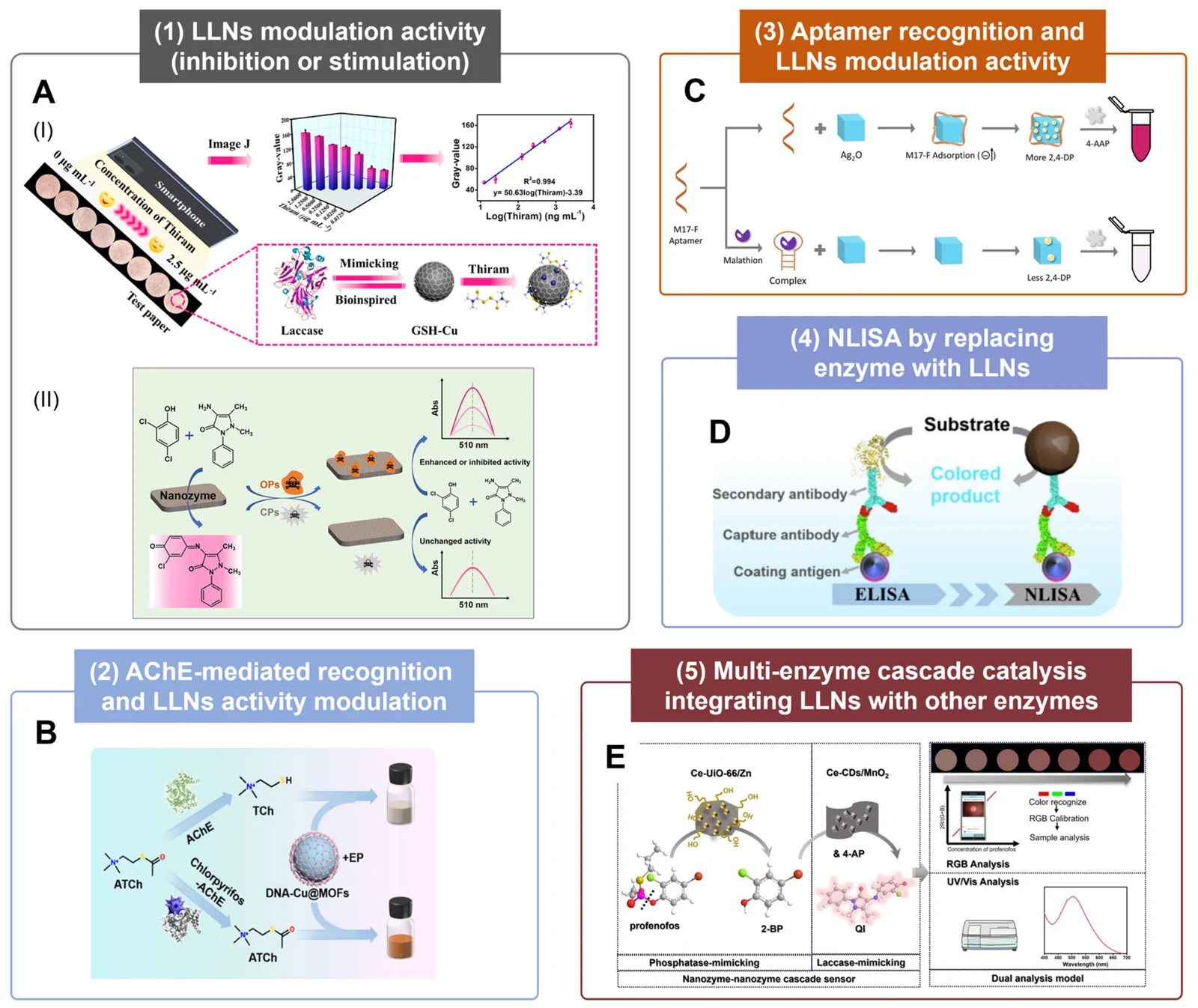
Five detection mechanisms employing LLNs for pesticide residue sensing. (**A**) Schematic diagrams of a GSH-Cu-based test paper for on-site detection of thiram and smartphone-assisted sensor array integrated with four LNNs for identification of OPs from CPs. (**I**) Adapted with permission from Ref. [56]. Copyright 2022 Elsevier. (**II**) Adapted with permission from Ref. [114]. Copyright 2023 Elsevier. (**B**) Reaction mechanism of chlorpyrifos detection based on AChE recognition and DNA-Cu@MOFs. Adapted with permission from Ref. [83]. Copyright 2025 Elsevier. (**C**) Schematic diagram of the colorimetric biosensor for malathion detection based on aptamer recognition and Ag_2_O. Adapted with permission from Ref. [92]. Copyright 2024 Elsevier. (**D**) Schematic representation of NLISA by replacing enzymes with LLNs. Adapted with permission from Ref. [58]. Copyright 2022 Elsevier. (**E**) Cascade sensing platform for ultrasensitive profenofos detection. Adapted with permission from Ref. [26]. Copyright 2025 Elsevier.

Generally, five detection mechanisms are employed (Figure 9), including (1) LLN modulation activity (inhibition or stimulation), (2) AChE-mediated recognition and LLN modulation activity, (3) aptamer recognition and LLN modulation activity, (4) nanozyme-linked immunosorbent assay (NLISA) by replacing the enzyme with LLNs, and (5) multi-enzyme cascade catalysis, integrating LLNs with other enzymes.

To date, a variety of LLNs have been developed for pesticide detection, operating on the principle that their activity is modulated upon exposure to target analytes. As shown in Figure 9A(I), a GSH-Cu LLN enables sensitive, selective thiram sensing, wherein thiram passivates the surface, inhibits laccase-like activity, weakens chromogenic substrate adsorption, and thus alters output color [56]. Smartphone-based image analysis enabled the conversion of colorimetric responses into quantitative data, allowing on-site detection of thiram over a linear range of 2.5–250 ng mL^−1^ with an LOD of 2.00 ng mL^−1^. Through rational design of MOF precursors, an electroactive LLN (Fe_3_O_4_/Cu-MOF) was engineered for ratiometric electrochemical detection of thiram [77]. Based on analyte-induced catalytic activity inhibition and conductivity enhancement of Fe_3_O_4_/Cu-MOF, thiram in fruits and vegetables was sensitively and selectively detected. Ligand substituent engineering had been demonstrated to be an effective strategy for enhancing the laccase-like activity of Cu-BDC-NO_2_, which was integrated into hydrogel kits to fabricate a portable platform for the selective on-site glyphosate detection [178]. A bioinspired LLN (Cu@COP) was constructed and used for thiram detection in fruit and vegetable samples [12]. Mechanistically, Cu@COP cleaved the S-S bond of thiram and formed thiram-Cu@COP complexes, thereby enhancing the laccase-like activity of Cu@COP. The analyte-triggered activity amplification was applied to construct a highly sensitive and selective colorimetric assay, enabling the reliable on-site monitoring of thiram residues. A colorimetric sensing array based on Cu–amino acid coordination complexes enabled pesticide discrimination via pattern recognition [33]. Integration with YOLOv8 deep-learning automated classification [181,182,183] and enhanced throughput, a Cu/Ce-MOF three-channel array, leveraging multienzyme-like activities, further discriminated six pesticides with 100% accuracy at 0.5 μg/mL [179].

The conventional analytical method for pesticide detection is the enzyme inhibition method based on natural acetylcholinesterase (AChE) and butyrylcholinesterase (BuChE) [184]. Generally, AChE and BuChE are considered as high-affinity biorecognition elements for organophosphorus pesticides (OPs) and carbamate pesticides (CPs). However, the enzyme inhibition method has several major drawbacks, such as high cost and poor stability of AChE/BuChE, narrow applicability, and limited discrimination performance. To avoid interference from CPs with similar AChE inhibitory effects, Song developed a selective recognition method for OP detection based on the differential modulation of four LLN activities in response to pesticides (Figure 9A(II)) [114]. Demonstrating excellent interference-free performance, this sensing array could accurately identify and discriminate between various OPs, even in the presence of interfering CPs. Coupled with a smartphone, this system successfully achieved the rapid and portable detection and discrimination of OPs in real food samples. To expand applicability, an integrated array that includes the Mel-Cu nanozyme and AChE, with complementary recognition capabilities, was constructed [46]. Combined with classification and regression algorithms of machine learning, this proposed integrated sensor array and predictive model enabled intelligent recognition of multi-category pesticides. To break pH limitations inherent to AChE inhibition methods, AChE was integrated with a robust LLN (DNA-Cu@MOFs), with AChE serving as the recognition element and DNA-Cu@MOFs as the signal amplification unit for the selective detection of OPs [83]. As shown in Figure 9B, thiocholine (TCh), generated from the AChE-catalyzed hydrolysis of acetylthiocholine (ATCh), inhibits the catalytic activity of DNA-Cu@MOFs, whereas chlorpyrifos inhibits AChE activity, thereby reducing TCh production and relieving the inhibition of DNA-Cu@MOFs. The resulting colorimetric sensing platform exhibited a low LOD of 0.75 ng mL^−1^ for chlorpyrifos and satisfactory recoveries of 97.2–107.9% in food samples, demonstrating high sensitivity, selectivity, and reliability.

Recent studies have advanced LLN-based sensor arrays for the discrimination of muti-category pesticides. A sensor array was developed using the Asp-Cu nanozyme with laccase-like, peroxidase-like, and superoxide dismutase-like activities as the sole sensing unit [48]. Based on the modulated variations of different pesticides in multiple activities, this sensor array simultaneously distinguished multiple categories of pesticides in real food samples. Using the established concentration-independent model, blind samples with broad-spectrum pesticides were accurately identified. To circumvent the susceptibility of single-signal colorimetric arrays to background interference, a multi-signal sensor array was constructed using a fluorescent LLN (Cu-BDC-NH_2_), enabling the cross-reactive discrimination of broad-spectrum pesticides [101]. One system utilized three nanozymes with laccase- and peroxidase-like activities to discriminate sulfonylurea residues, with a predictive model reporting 100% accuracy in real food samples [117]. Another platform employed two nanozymes exhibiting four enzyme-mimicking activities to classify multiple pesticide categories, and its integration with machine learning enabled concentration-independent identification with high accuracy [120]. A more recent study described a machine-learning-assisted multichannel nanozyme sensor array for simultaneous detection of pesticides and their metabolites, aiming to provide robust tracking and tracing of pesticide residues [119]. Critically, LLN-based sensor arrays offer a versatile, low-cost, and potentially portable platform for food safety analysis. Their strengths include enzyme-mimicking stability, multienzyme-like channel design, and compatibility with machine learning, and they demonstrated great potential in the detection and discrimination of pesticides in complex food matrices. However, their analytical maturity is constrained by non-specific sensing mechanisms, matrix susceptibility, limited sensitivity, reproducibility challenges, small training datasets, and insufficient validation against regulatory-grade methods. Future progress requires mechanistic studies, standardized synthesis and assay protocols, larger and more diverse datasets, lower LOD, and rigorous field validation.

In addition to AChE and BuChE, aptamers are another type of high-affinity biorecognition elements for pesticides [185]. Based on the fact that specific aptamers can preferentially bind to the target pesticide and enhance the laccase-like activities of Ag-based LLNs, Wu research groups constructed various aptamer-regulated colorimetric biosensors for the highly selective detection of malathion, λ-cyhalothrin and acetamiprid [66,92,94]. As shown in Figure 9C, the M17-F aptamer enhances Ag_2_O NPs activity by adsorbing onto the surface, promoting 2,4-DP oxidation to semiquinone radicals that react with 4-aminoantipyrine (4-AAP) to produce a colorimetric signal. Malathion preferentially binds to the aptamer, thereby suppressing the aptamer-induced enhancement of Ag_2_O NP activity and enabling quantitative detection [83]. The resulting colorimetric biosensor achieved an LOD of 5.85 nM for malathion detection. When integrated with smartphone-based readout, these aptamer-regulated sensing platforms demonstrated satisfactory performance in real food samples, highlighting their potential for reliable, portable, and on-site food safety monitoring.

The enzyme-linked immunosorbent assay (ELISA) is another conventional tool for pesticide detection. However, its practical application is limited by the poor stability of natural enzymes under harsh conditions. To address this limitation, a nanozyme-linked immunosorbent assay (NLISA) was developed, in which a LLN, His–Cys–Cu, was employed as a substitute for natural enzymes (Figure 9D) [58]. Leveraging the excellent laccase-like activity and utilization efficiency of His–Cys–Cu, NLISA showed a 150-fold improvement in sensitivity compared with the conventional ELISA, achieving an IC_50_ of 133 pg mL^−1^ for imidacloprid. Furthermore, NLISA exhibited selective and reliable detection of imidacloprid in complex food matrices, including apple juice, milk, and tea. This study underscores the considerable potential of NLISA to incorporate LLNs for applications in the food industry.

Single nanozyme-based colorimetric sensors often suffer from weak signals and poor specificity. Multi-enzyme cascade catalysis overcomes these issues by amplifying signals and enhancing intermediate utilization, enabling sensitive and selective detection of target pesticide. A bi-enzyme cascade platform integrating alkaline phosphatase and a LLN (ATP@ [Ru(bpy)_3_]^2+^) was designed for pyrethroid detection [121]. Coupling the colorimetric-fluorescence dual-mode sensing with machine learning, this method achieved on-site and intelligent detection of pyrethroids in real samples. A cascaded amplification sensing strategy was established for profenofos detection using Ce-UiO-66/Zn and Ce-CDsMnO_2_ [26]. Based on the phosphohydrolase-like activity of Ce-UiO-66/Zn and laccase-like activity of Ce-CDs/MnO_2_, profenofos was enzymatically transformed into a colored product for colorimetric detection (Figure 9E). Integrated with a smartphone platform, this method achieved recoveries of 84.7–99.8% in food matrices, with a limit of detection of 14.6 ng mL^−1^, enabling sensitive, selective, on-site monitoring of profenofos.

Collectively, activity-modulation assays offer operational simplicity, but may be affected by non-specific inhibitors in food matrices. AChE- and aptamer-assisted systems provide improved molecular recognition, although their performance depends on the stability and affinity of the biological recognition elements. NLISA and multi-enzyme cascade platforms provide substantial signal amplification, but introduce additional preparation steps and potential sources of batch-to-batch variability. Therefore, improvements in sensitivity should be evaluated together with selectivity, recovery, fabrication reproducibility, and performance in real food samples.

#### 4.2.3. Antibiotic Residue Detection and Elimination

To ensure food safety and protect public health, it is of great significance to develop rapid and accurate detection technologies, precise and intelligent recognition methods, and efficient removal strategies for antibiotics [186,187]. As shown in Table 4, LLNs have been widely applied for the detection, discrimination and elimination of multi-category antibiotics, such as β-lactam antibiotics, tetracycline antibiotics, and aminoglycoside antibiotics.

**Table 4:** Applications of LLNs toward antibiotic residue detection and elimination in various food matrices.

Antibiotics	Food Sample	LLNs	Detection Modes	Performances	Refs.
Oxytetracycline	Chicken breast, pork, honey	CuNZs	Colorimetric	LOD: 0.148 μM, recovery rate: 88.72–108.14%	[188]
Oxytetracycline and tetracycline	Milk, honey, chicken	CeCo-MOF	Colorimetric	LOD: 1.559 nM and 1.891 nM, recovery rate: 92.21–108.01%	[81]
Tetracycline	Tap water, cucumber, apple	Ce-UiO-66, Ce-MOF-808	Colorimetric	LOD: 1.83 ng/L and 2.75 ng/L, removal efficiencies: 50.4% and 75.38%	[80]
Kanamycin	Tap water, milk, honey	Cu-BH	Dual-mode (colorimetric & fluorescence)	LOD: 0.033 nM and 0.26 nM, recovery rate: 96.00–104.15%	[53]
	Tap water, honey, pork	Ag_2_O NPs	Colorimetric	LOD: 0.13 nM, recovery rate: 88.57–97.60%	[91]
	Water, honey, milk, pork	Mn-Car	Colorimetric	LOD: 0.102 μM, recovery rate: 83.3–100.5%	[189]
β-lactam antibiotics (e.g., ampicillin, penicillin)	Honey, egg	Cu-BTC@His	Colorimetric	Discrimination accuracy rate: 95.92%	[75]
	Pork, beef	Cu-MOF	Dual-mode (colorimetric & fluorescence)	LOD: 0.13 μM	[190]
Kasugamycin	Meat, cucumber, milk powder	Ag_2_O	Colorimetric	LOD: 2.8 nM	[191]
Five antibiotics	Chicken	GMP-Cu NAD-Cu Arac-Cu	Colorimetric	LOD: 2 μM, discrimination accuracy rate: 100%	[40]
Quinolone antibiotics	Honey, egg	Cu_2_(OH)_2_SO_4_	Colorimetric	Discrimination accuracy rate: 91.95%	[116]
Multi-category antibiotics	Honey	CA-Cu	Colorimetric	Discrimination accuracy rate: 97.70%	[115]

**Figure 10:**
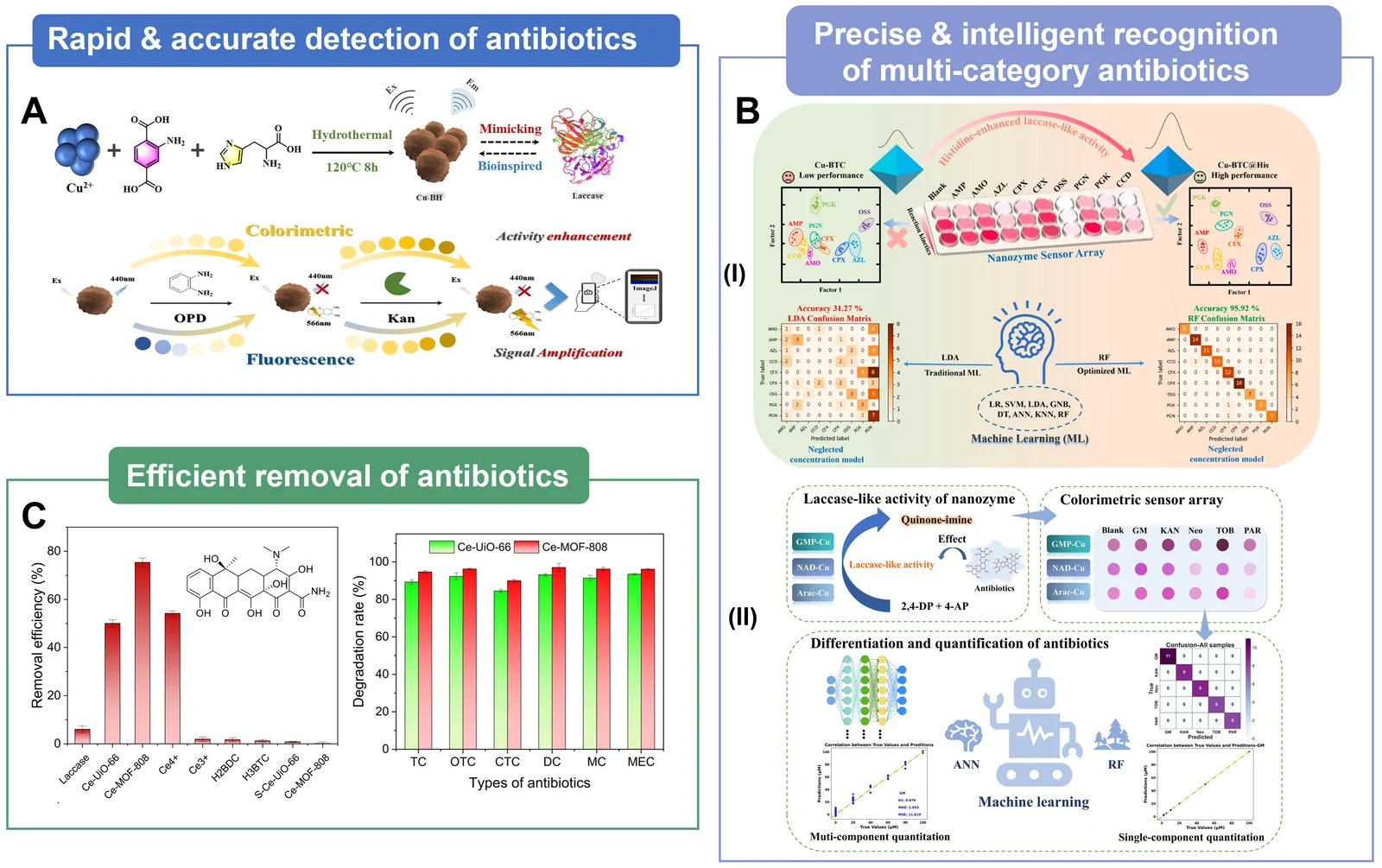
Applications of LLN-based method for antibiotic residue detection and elimination. (**A**) Schematic illustration of a multi-modal sensing strategy based on Cu-BH for kanamycin analysis. Adapted with permission from Ref. [53]. Copyright 2023 Elsevier. (**B**) Schematics of sensor arrays based on LLNs for the detection of antibiotics combined with machine learning algorithms. (**I**) A sensor array based on Cu-BTC@His nanozyme for the recognition of beta-lactam antibiotics. Adapted with permission from Ref. [75]. Copyright 2025 Elsevier. (**II**) A sensor array based on three copper-based nanozymes for the detection of aminoglycoside antibiotics. Adapted with permission from Ref. [40]. Copyright 2025 Elsevier. (**C**) Comparison of TC removal efficiency and degradation rates of multiple antibiotics catalyzed by Ce-UiO-66 and Ce-MOF-808. Adapted with permission from Ref. [80]. Copyright 2025 Elsevier.

Rapid and accurate detection of antibiotics. By virtue of the inhibition effect of antibiotics toward the enzymatic activity of LLNs, various colorimetric sensor arrays have been established for the simultaneous detection and discrimination of multiple antibiotics. A fast and simple colorimetric assay for oxytetracycline (OTC) was established, based on the laccase-like activity of CuNZs [188], which catalyzed OTC to yield a yellow product with concentration-dependent absorbance, enabling successful OTC detection in chicken, pork and honey samples. A CeCo-MOF-based colorimetric sensor array was developed for TCs, in which Ce coordinates the unique β-diketone structure of TCs, while Co binds their acetamide and β-diketone groups [81]. The reliability and practicability of this method were confirmed by excellent anti-interference performance, satisfactory discrimination and recovery rate of TCs in actual sample tests, and 96.67% recognition accuracy for blind samples. To enhance the selectivity and sensitivity, a multi-mode detection strategy for kanamycin was established, based on the laccase activity and fluorescence properties of the Cu-BH nanozymes [53]. As illustrated in Figure 10A, the presence of kanamycin enables the activity enhancement and signal amplification of Cu-BH, facilitating sensitive detection in water, milk and honey, while the smartphone-based RGB readout facilitates visual analysis. In addition to copper-based LLNs, a non-copper-based LLN (cubic Ag_2_O nanoparticles), whose activity is effectively inhibited by kanamycin, was adopted to construct a colorimetric sensor for the rapid detection of kanamycin [91]. More recently, another non-copper-based multivalent LLN, Mn-Car, was used to develop a colorimetric platform for ultrasensitive and selective detection of kanamycin in food [189].

Precise and intelligent recognition of multi-category antibiotics. Histidine engineering was applied to enhance the laccase-like activity of the Cu-BTC nanozyme [75]. As illustrated in Figure 10B(I), β-lactam antibiotics inhibit Cu-BTC@His activity in a time-dependent manner, enabling the construction of a three-channel, kinetics-based nanozyme sensor array for discriminating nine β-lactams. Integration with machine learning further improved the accuracy, to 95.92% in honey samples. Similarly, a colorimetric sensor array was developed for sensing aminoglycoside antibiotics, based on three LLNs (Figure 10B(II)) [40]. Integrating this with the ANN and RF algorithms, the discrimination of five aminoglycoside antibiotics in real food matrices was successfully achieved. These LLNs-based sensor arrays provide great promise for the rapid and intelligent recognition of various antibiotics.

Efficient removal of antibiotics. Mimicking the catalytic active site of multicopper laccase, two Ce^4+/^Ce^3+^-based LLNs (Ce-UiO-66 and Ce-MOF-808), which are capable of degrading tetracycline antibiotics (TCs), were constructed [80]. Compared to natural laccase (from *Trametes versicolor*), Ce-UiO-66 and Ce-MOF-808 showed higher removal efficiencies of tetracycline than their counterparts, and excellent degradation rates (>80%) for multiple antibiotics (Figure 10C). Mechanistically, Ce-UiO-66 and Ce-MOF-808 can remove multiple TCs by oxidative degradation via the non-radical electron-transfer mechanism.

#### 4.2.4. Food Additives

Food additives are indispensable components in the modern food industry. They play critical roles in improving food quality, extending shelf life and enhancing processing efficiency. However, the overuse of food additives has exacerbated food safety risks. The quantitative analysis of food additives is an important part of food safety control. In recent years, LLNs have attracted considerable attention as promising candidates for the detection of food additives (Table 5).

**Table 5:** Applications of LLNs toward food additive detection in various food matrices.

Food Additives	Food Sample	LLNs	Detection Modes	Performances	Refs.
Sulfide	Baking soda, rock sugar, konjac flour, and xylitol	GMP-Cu	Colorimetric	LOD: 0.67 μM, recovery rate: 97.40–104.85%	[37]
	Wine, egg, and milk	Cu_2_(OH)_3_NO_3_	Colorimetric	LOD: 5 μM, recovery rate: 99.2–107.4%	[118]
	Rock sugar, xylitol, and baking soda	MA-Cu	Colorimetric	LOD: 18.81 nM, recovery rate: 92.53–109.48%	[45]
Sulfite	Red wine	AlgCu	Colorimetric	LOD: 0.78 μM, recovery rate: 96–106%	[192]
Thymol	Honey	CuGaa	Colorimetric	LOD: 7.4 μM, recovery rate: 94.73–103.95%	[193]
(+)-Catechins	Milk and milk powder	MI-Bpy-Cu	Colorimetric	LOD: 0.83 μg/mL, recovery rate: 95.27–104.87%	[194]

**Figure 11:**
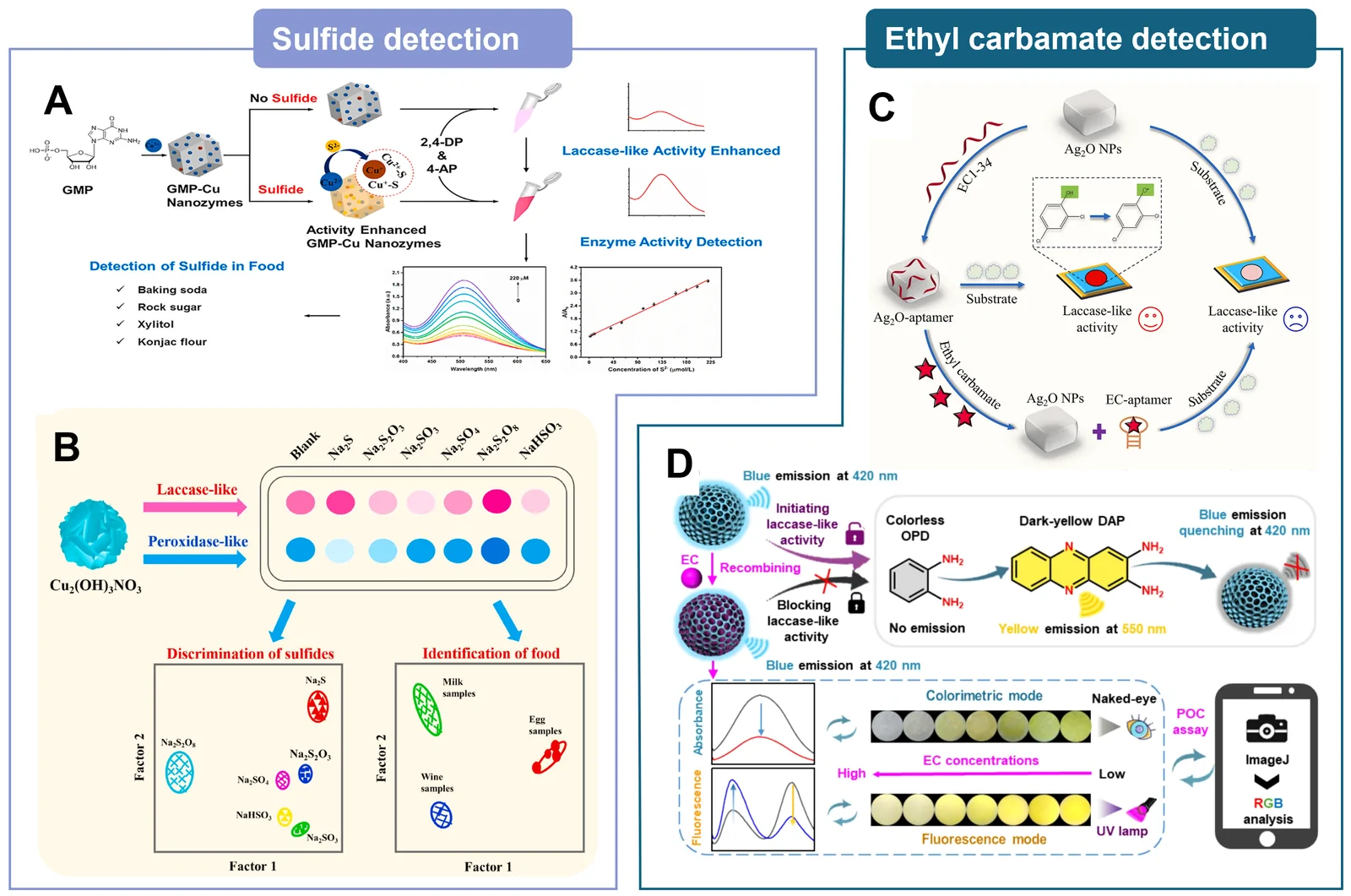
Applications of LLN-based methods in the detection of food additives and other hazardous candidates. (**A**) Strategy of sulfide detection based on GMP-Cu nanozymes. Adapted with permission from Ref. [37]. Copyright 2021 Elsevier. (**B**) Schematic illustration of nanozyme sensor array for sulfide discrimination. Adapted with permission from Ref. [118]. Copyright 2024 Elsevier. (**C**) Schematic diagram of EC detection process using Ag_2_O-aptamer hydrogel. Adapted with permission from Ref. [93]. Copyright 2025 Elsevier. (**D**) Schematic diagram of an EC-switchable MIP-encapsulated laccase-like Cu-BH@MIP-based onsite fluorescence/colorimetric dual-mode self-calibrating assay for EC residues. Adapted with permission from Ref. [102]. Copyright 2025 Elsevier.

Sulfides are widely used as food preservatives and flavor regulators. However, chronic overexposure poses health risks, necessitating the development of simple, efficient, and specific detection methods. LLN exhibit sulfide-enhanced activity through Cu–S bond formation, with the degree of enhancement showing a linear correlation with S^2−^ concentration. This amplification-based sensing strategy offers advantages such as reduced background signal, improved anti-interference capability, enhanced stability, and greater selectivity and sensitivity. As shown in Figure 11A, a selective colorimetric approach using GMP–Cu nanozymes with laccase-like activity has been developed, enabling sulfide detection in various food matrices based on this linear activity–concentration relationship [37]. Similarly, leveraging the distinct regulatory effects of different sulfides on Cu_2_(OH)_3_NO_3_ catalysis (Figure 11B), a nanozyme sensor array was constructed for discriminating six sulfide species in complex food samples such as wine, egg, and milk [118]. Moreover, the developed Cu_2_(OH)_3_NO_3_-based sensor had higher sensitivity than the classical iodometric method and lower LOD than other nanozymes. Using the alginate–copper laccase nanozyme AlgCu, the rapid colorimetric detection of sulfite in red wine was realized [192]. By integrating the colorimetric assay with smartphone color readouts, a sulfite concentration down to 4.9 μM could be detected, indicating excellent suitability for a point-of-need application in wine quality control. More recently, a MA–Cu-based colorimetric method has been established for sulfide detection in food additives including rock sugar, xylitol, and baking soda [45].

(+)-Catechin and thymol are commonly utilized as food additives, due to antibacterial properties. However, high concentrations of (+)-Catechin and thymol can induce health risks such as dizziness and nausea, underscoring the urgent need for simple, efficient, and specific detection techniques. To address this concern, a colorimetric sensing strategy was established using a unique LLN (MI-Bpy-Cu) for (+)-Catechin detection [194]. This method had wide linear range (0–280 μg/L), low LOD (0.83 μg/L), good anti-interference and high selectivity, demonstrating considerable potential for (+)-Catechin detection in dairy products. A CuGaa-based colorimetric method integrated with logic-gate technology was developed for the rapid detection of thymol residues in honey [193]. The constructed LLN-based sensing strategy had good selectivity and interference resistant ability, with desirable recovery rates (95–104%) in honey samples.

#### 4.2.5. Other Hazardous Analytes

In addition to mycotoxins, pesticides, antibiotics and food additives, a variety of hazardous substances such as ethyl carbamate, allergenic proteins and bisphenol A also endanger food safety. LLNs have been extensively utilized for the detection of these hazardous analytes in recent years (Table 6).

**Table 6:** Applications of LLNs toward other hazardous analytes detection in various food matrices.

Target Analytes	Food Sample	LLNs	Detection Modes	Performances	Refs.
Ethyl carbamate	Chinese Baijiu	Cubic Ag_2_O	Colorimetric	LOD: 10 μg/L, recovery rate: 91.2–100.9%	[93]
	Chinese Baijiu, Huangjiu, and cooking wine	Cu-BH@MIP	Dual-mode (Colorimetric & fluorescence)	LOD: 3.06 μg/L and 2.53 μg/L, recovery rate: 99.3–102.1%	[102]
Allergenic proteins	Milk, yogurt, candy, and infant formulas	LM nanozymes	Colorimetric	LOD: 0.056 ng/mL, recovery rate: 96.5–101.2%	[55]
Protamine and trypsin	Milk	Bpy-Cu	Colorimetric	LOD (protamine): 3.06 μg/L, LOD (trypsin): 3.3 ng/mL	[47]
Spermine	Tap water, corn and pork	Mn_3_O_4_ NPs	Colorimetric	LOD: 0.094 μg/mL, recovery rate: 88.6–95.3%	[195]
Histamine	Pork, fish and shrimp	AgCit	Colorimetric	LOD: 27 μg/mL, recovery rate: 89.9–96.8%	[196]
	Fish and shrimp	Mn_3_O_4_ NPs	Colorimetric	LOD: 0.148 μg/mL, recovery rate: 85.7–95.1%	[197]
Bisphenol A	Infant juice package	Zr-TGA	Colorimetric	LOD: 1.67 μM, recovery rate: 98.3–106.3%	[57]
		GA-CuAsp	Colorimetric	LOD: 0.75 μM, recovery rate: 96.2–105.6%	[49]
	Milk powder	Zein-Cu	Colorimetric	LOD: 0.80 μM	[32]
Diethylstilbestrol	Milk	CeO_2_@CuTbDPA	Colorimetric	LOD: 1 pM	[198]
Copper ions	Alcoholic beverage	PDA/MnOx	Colorimetric	LOD: 61 nM	[199]

Ethyl carbamate (EC), a nitrogenous toxic compound commonly generated as a by-product in alcoholic beverages and fermented foods, exhibits neurotoxic and carcinogenic properties [108]. To mitigate the risks associated with EC residues, developing accurate, rapid and simple analytical methods is of great importance for on-site, point-of-care early warning and traceability analysis. To improve the selectivity of LLNs toward EC, various recognition elements such as aptamer and molecular imprinted polymer (MIP) have been employed [200]. A portable colorimetric paper-based sheet was developed for EC detection in Chinese Baijiu by exploiting the aptamer-binding-induced enhancement of the laccase-like activity of cubic Ag_2_O NPs [93]. As illustrated in Figure 11C, The EC1-34 aptamer warped on the surface of Ag_2_O NPs, forming a negatively charged recognition probe, which is subsequently immobilized onto a carrier membrane. In the absence of EC, enhanced activity turns the sensor red. Upon EC introduction, aptamer-EC binding reduces catalytic activity, yielding a pink color. A molecularly imprinted, switchable LLN (Cu-BH@MIP) was developed for the dual-mode (colorimetric/fluorescence) self-calibrated determination of EC residues [102]. Competitive EC binding to Cu-BH@MIP cavities regulates catalytic oxidation of OPD to yellow DAP, enabling simultaneous colorimetric and fluorescence readouts (Figure 11D). On this basis, a smartphone-assisted portable device enabled visual EC quantification, with its selectivity and reliability validated in alcoholic beverages (Baijiu, Huangjiu, and cooking wine). In summary, these LLN-based sensing methods enable the sensitive, reliable, and real-time detection of EC in fermented foods.

Sensitive and reliable detection of food allergens and biogenic amines, especially in cross-contaminated foods, is critical for effective food-safety risk management. A laccase mimic (LM) was employed as the catalytic label in a nano-ELISA for alpha-lactalbumin (α-LA), and a smartphone-integrated portable system was further developed for rapid on-site analysis of α-LA, achieving a low LOD (0.056 ng/mL) and acceptable recovery rates (96.5–101.2%) in food samples [55]. A smartphone-integrated colorimetric sensor based on a LLN (Mnf) was established for spermine detection [195]. Positively charged spermine induces the aggregation of negatively charged Mnf via electrostatic interactions, reducing surface area and active sites, thereby inhibiting catalytic activity. This inhibition enables rapid, highly selective spermine quantification, offering a practical tool for food safety monitoring. Employing the enhanced laccase-mimic activity of AgCit, wherein the H2 aptamer increased the affinity of AgCit to 2,4-DP to promote semiquinone radical generation, a colorimetric aptasensor was designed for sensitive (LOD: 27 μg/L) and reliable (recovery rate: 89.9–96.8%) detection of histamine in food [196]. Based on Cu^2+^-mediated laccase-mimic activity of Mnf, a switching off–on colorimetric sensor for rapid and selective detection of histamine was constructed [197]. Specifically, Cu^2+^ initially suppressed the Mnf-catalyzed oxidation of 2,4-DP, preventing color development. In the presence of histamine, Cu^2+^ was preferentially consumed via coordination with histamine, thereby restoring the laccase-like activity of Mnf and enabling an off–on switching response. This sensor achieved sensitive, reliable and on-site detection of histamine in fish and shrimp, providing a reliable alternative for the rapid monitoingr of histamine in food samples.

Beyond conventional food contaminants, bisphenol A (BPA) from plastic packaging poses emerging risks due to its endocrine-disrupting effects, yet its trace detection remains challenging [201]. LLNs provide a highly sensitive and selective alternative strategy for the detection of BPA. A thioglycolic acid-modified MOF-818 LLN (ZrTGA) with enhanced laccase-like activity was prepared and integrated into a smartphone-based logic-gate assay for rapid BPA quantification in infant juices [57]. Similarly, gallic acid-activated CuAsp (GA-CuAsp) enabled sensitive colorimetric detection of BPA with improved selectivity and interference resistance, reducing the LOD to 0.75 µM in infant-food packaging [49]. Two LLNs, Ce-UiO-66 and Ce-MOF-808, exhibited considerable activity in the oxidative degradation of BPA, significantly outperforming natural laccase (from *Trametes versicolor*) [79]. More recently, Zein-Cu, which exhibits markedly enhanced catalytic activity and superior stability compared to natural laccase (from *Trametes versicolor*), has demonstrated efficient degradation of plastic food packaging [32]. More recently, a colorimetric assay based on PDA/MnOx nanoparticles was reported for Cu^2+^ detection, achieving a low detection limit of 61 nM over a wide linear range (0.5–100 μM) [199]. When integrated with a smartphone, this PDA/MnOx-based colorimetric assay enabled instrument-free on-site analysis, offering a promising strategy for beverage-safety monitoring.

## 5. Conclusions and Future Perspectives

LLNs hold immense promise for food quality and safety monitoring, owing to their robustness, cost-effectiveness, structural tunability, exceptional sensitivity, and compatibility with diverse detection modalities. Employing both bioinspired and structural engineering strategies, a diverse array of LLNs has been constructed, utilizing coordination engineering, metal-based nanomaterials, and hybrid functional nanostructures. These design strategies enable the regulation of active sites, electronic structures, and the catalytic microenvironment, thereby enhancing the catalytic performance and analytical capabilities of LLNs. By combining LLNs with advanced sensing technologies such as colorimetric, fluorescent, electrochemical, and multimodal sensing platforms, the rapid, sensitive and reliable detection of food quality indicators and safety risk factors have been achieved. LLN-based sensing systems have demonstrated great potential for applications in detecting bioactive substances, food freshness, food classification, and harmful substances such as mycotoxins, pesticides and antibiotics residues, and other hazardous chemicals.

Despite the rapid progress in LLN-based sensing, several fundamental issues remain unresolved. In many reported systems, the active-site structures and catalytic pathways are inferred primarily from indirect characterization or theoretical calculations, and direct experimental evidence remains limited. In addition, differences in substrate selection, pH, temperature, kinetic models, and activity units hinder meaningful comparison across studies. Establishing standardized catalytic evaluation protocols and reporting requirements is therefore essential. Batch-to-batch reproducibility, long-term storage stability, matrix-specific validation, and comparison with established analytical methods should also be considered before LLN-based platforms can advance from proof-of-concept studies to practical food analysis.

Future endeavors toward LLN-based sensors should prioritize the enhancement of catalytic efficiency, selectivity, and specificity, particularly to mitigate matrix effects in complex food matrices. Advancing multi-enzyme cascade systems alongside multimodal detection platforms, integrated with smartphone-based readouts, will be instrumental in enabling reliable on-site sensing. To address current limitations in fabrication complexity, environmental stability, and cost, sustainable and scalable synthesis routes must be developed. Furthermore, the incorporation of computational modeling and machine learning holds great promise for accelerating material design, optimizing sensor performance, and enabling real-time, automated decision-making. Equally critical is the need to comprehensively evaluate toxicity and biocompatibility in food-related contexts, alongside proactive engagement with regulatory frameworks, to ensure safety and compliance. Ultimately, interdisciplinary collaboration among materials scientists, data analysts, and food safety regulators will be essential to translate these advanced LLN-based systems from laboratory innovations into practical, deployable solutions that are both economically viable and environmentally sustainable. With these ongoing efforts, LLNs are anticipated to serve an increasing vital role in food quality and safety monitoring.

## Figures and Tables

**Table 1:** Applications of laccase-like nanozymes in food quality monitoring in various food matrices.

Target Detection	Food Sample	LLNs	Detection Mode	Performance	Ref.
Phenolic compounds	Coffee, tea, onions and guava	N-doped MnO_2_ nanoflowers	Colorimetric	LOD: 0.353 μM	[25]
	Juice, beer, and wine	GA-Cu	Colorimetric	Linear range: 1–100 μM, discriminant accuracy: 100%	[50]
	Black tea, honey, and grape juice	VA-Cu	Colorimetric	Discriminant accuracy: 100%	[51]
	Black tea, coffee, and wine	Cu-BTC	Dual-mode (colorimetric and photothermal)	Discriminant accuracy: 100%	[76]
	Beverage and honey	Co-MOF	Colorimetric	LODs for catechol, hydroquinone, resorcinol, chlorogenic acid, vanillic acid: 8.8 nM, 9.5 nM, 10 nM, 9.5 nM, 10 nM	[63]
	Sunflower oil, mayonnaise, and butter	CUO@Pd	Colorimetric	Discriminant accuracy: 100%	[60]
	Honeysuckle	Fe-KAC	Dual-mode (electrochemical & colorimetric)	LOD: 0.0072 μM	[143]
Catechols	Fruit juice	CYP-Mnzyme	Colorimetric	LOD: 0.5 μM	[144]
Tannic acid	Brandy	Fe@CN-20	Colorimetric	LOD: 0.13 mg/L, linear range: 5–100 mg/L, recovery rate: 99–111%, RSD < 3%	[24]
	Brandy, white liquor, and tea beverages	VCo-N-C	Colorimetric	LOD: 0.032 mg/L, linear range: 0.1–3000 mg/L, recovery rate: 99–102%, RSD < 2%	[87]
Bioactive compounds (resveratrol, tea polyphenol, chlorogenic acid, rutin, and vanillin)	Coffee, juice and wine	A-Cu G-Cu C-Cu T-Cu	Colorimetric	Linear range: 1.5–150 ng/mL, LOD: 0.42 ng/mL	[13]
Quercetin	Green pepper, dill, and red onion	Cu-TA NShs	Colorimetric	Linear range: 0.35–32.09 μM, LOD: 0.42 μM	[145]
	Fruit juice	Nitrogen/nickel	Electrochemical	Linear range: 3.4 nM, LOD: 0.064 ng/mL	[106]
Flavonoids (apigenin, kaempferol, silymarin, catechin and luteolin)	Chinese wolfberry	CA-Cu	Colorimetric	LOD: 1 μM, recovery rate of luteolin: 98.8–104.0%, discriminant accuracy: 100%	[52]
Total antioxidant capacity (ascorbic acid)	Commercial beverages	CuNi/CoMoO_4_	Colorimetric	Linear range: 1–150 μM, LOD: 0.7 μM	[16]
	Fruit juices	CeO_2_	Colorimetric	Linear range: 0.5–50 μM, LOD: 0.67 μM, recovery rate: 95–104%	[22]
Total antioxidant capacity (EGCG)	Tea	MnO_2_@Ag	Colorimetric	LOD: 1 μM	[146]
Guaiacol	4 juices	CuN_4_-G	Electrochemical	Linear range: 5–50,000 pM, LOD: 1.2 pM, recovery rate: 94–106%	[85]
Elimination and detection of phenolic compounds	Juice	AMP-Cu	Colorimetric	Linear range: 0.1–100 μM, LOD: 0.033 μM	[35]
Meat freshness	Pork, beef and cod	MnZn-MOF	Colorimetric	Discriminant accuracy: 100%	[147]
Tea discrimination	Tea	Cu-ATP Cu-ADP Cu-AMP	Colorimetric	Discriminant accuracy: 100%	[38]
Adulteration identification	White peony tea	NAD-Cu CuNA	Colorimetric	Discriminant accuracy: 100%	[39]

## Data Availability

No new data were created or analyzed in this study. Data sharing is not applicable to this article.

## References

[1] Wang Q., Zabed H.M., Zhao M., Qi X. (2025). A Deeper Insight into the Thermostability of a Novel Laccase Designed by Data-Driven Mining and Rational Engineering. J. Agric. Food Chem..

[2] He J., Li J., Wang Y., Wang Y., Wu P. (2025). Recent Progress on the Rational Design of Laccase Mimics. Chem. Asian J..

[3] Zhou M., Fakayode O.A., Ren M., Li H., Liang J., Yagoub A.E.A., Fan Z., Zhou C. (2023). Laccase-catalyzed lignin depolymerization in deep eutectic solvents: Challenges and prospects. Bioresour. Bioprocess..

[4] Zhai T., Wang H., Dong X., Wang S., Xin X., Du J., Guan Q., Jiao H., Yang W., Dong R. (2024). Laccase: A Green Biocatalyst Offers Immense Potential for Food Industrial and Biotechnological Applications. J. Agric. Food Chem..

[5] Zhou M., Li C., Zhu Z., Huang L., Dai C., Zhang L., Fakayode O.A., Zhou C. (2025). Advances in catalytic conversion of lignin to vanillin using ionic liquids and deep eutectic solvents. Food Chem..

[6] Nazar M., Xu Q., Zahoor, Ullah M.W., Khan N.A., Iqbal B., Zhu D. (2023). Integrated laccase delignification with improved lignocellulose recalcitrance for enhancing enzymatic saccharification of ensiled rice straw. Ind. Crops Prod..

[7] Xu L.X., Sethupathy S., Liang Z., Zhuang Z.P., Zhang Y.W., Sun J.Z., Zhu D.C. (2024). NAD plus regeneration-coupled enzymatic bioconversion of lignin-derived vanillin into vanillic acid: A cleaner production approach. Ind. Crops Prod..

[8] Liaqat F., Xu L., Khazi M.I., Ali S., Rahman M.U., Zhu D. (2023). Extraction, purification, and applications of vanillin: A review of recent advances and challenges. Ind. Crops Prod..

[9] Yang R.Q., Liu Z.Y., Chen H.L., Zhang X.N., Sun Q., El-Mesery H.S., Lu W.J., Dai X.L., Xu R.J. (2025). Advances in Nanozyme Catalysis for Food Safety Detection: A Comprehensive Review on Progress and Challenges. Foods.

[10] Gao S.P., Chai X.Y., Zheng X.Y., Wang C.T., Zhang D., Zhang Y., Zhou R.Y., Zou X.B. (2026). Gold Nanozyme-Catalyzed SERS Detection of Food Contaminants. J. Agric. Food Chem..

[11] Ren X., Liu J., Ren J., Tang F., Meng X. (2015). One-pot synthesis of active copper-containing carbon dots with laccase-like activities. Nanoscale.

[12] Shi Y.D., Wang M.P., Wu H., Xiao S.J., Yue Z.Y., Yuan D.Z., Shao X.X., Yu F.T., Zhang L. (2025). Bioinspired copper-doped covalent organic polymers with laccase-mimicking activity for on-site monitoring of thiram in fruit and vegetable samples. Anal. Chim. Acta.

[13] Liu C., Huang Q. (2025). LTP-assisted fabrication of laccase-like Cu-MOF nanozyme-encoded array sensor for identification and intelligent sensing of bioactive components in food. Biosens. Bioelectron..

[14] Gao S., Sun Q., Katona J., Zhang D., Zhang Y., Zou X. (2025). Recent Advances in Metal-Organic Framework Nanozyme (MOFzyme)-Based Biosensors for Detecting Food Contaminants. J. Agric. Food Chem..

[15] Wang L., Liu Z., Yao L., Liu S., Wang Q., Qu H., Wu Y., Mao Y., Zheng L. (2024). A Bioinspired Single-Atom Fe Nanozyme with Excellent Laccase-Like Activity for Efficient Aflatoxin B(1) Removal. Small.

[16] Sun M., Dang Y., Du R., Lu Z., Wang Y., Wang X., Song C., Liu T., Wang G., Ye J. (2021). Experiment and theoretical insights into CuNi/CoMoO_4_ multi-functional catalyst with laccase-like: Catalysis mechanism, smartphone biosensing and organic pollutant efficient degradation. Chem. Eng. J..

[17] Zhang X., Zhang X., Lu Y., Huang Y. (2026). Recent advances in the analytical applications of laccase-like nanozymes. TrAC Trends Anal. Chem..

[18] Wang X., Li K., Han Q., Chen X., Hu J., Wang Y., Huang H., Li Y. (2026). Development of laccase-like nanozymes and the analytical applications in conjunction with emerging sensing technologies. Talanta.

[19] Wang Y., Hu J., Ma Y., Li K., Huang H., Li Y. (2024). Thiadiazol ligand-based laccase-like nanozymes with a high Cu(+) ratio for efficient removal of tetracyclines through polymerization. J. Hazard. Mater..

[20] Wu W., Peng C., Wang Y., Li J., Wang E. (2025). Building hydrophobic substrate pocket to boost activity of laccase-like nanozyme through acetonitrile-mediated strategy. J. Colloid Interface Sci..

[21] Ding L., Du M., Ping Q., Wang Y., Wang A., Huang R., Yu X. (2026). Design and Structural Regulation of Copper-based Laccase Nanozymes for Sensing Applications. J. Anal. Test..

[22] Yang Q., Cheng L., Yu J., Liu J., Liu J., Fan Y., Tang J., Huang S., Zhu X., Zhang Y. (2026). Sensitive “On-Off” Sensing Platform for Phosphate and Total Antioxidant Capacity Based on Phosphate-Mediated Cerium Oxide Laccase Nanozymes. J. Agric. Food Chem..

[23] Niu X., He H., Ran H., Wu Z., Tang Y., Wu Y. (2023). Rapid colorimetric sensor for ultrasensitive and highly selective detection of Fumonisin B1 in cereal based on laccase-mimicking activity of silver phosphate nanoparticles. Food Chem..

[24] Wang X., Liu H., Qiao C., Ma Y., Luo H., Hou C., Huo D. (2024). A dual-functional single-atom Fe nanozyme-based sensitive colorimetric sensor for tannins quantification in brandy. Food Chem..

[25] Chen Z., Li S., Yang F., Yue W. (2024). Construction of a colorimetric sensor array for the identification of phenolic compounds by the laccase-like activity of N-doped manganese oxide. Talanta.

[26] Chen M., Li X., Li Q., Li J., Gu Y., Yang P., Yang B., Yang Y. (2025). Construction of phosphatase- and laccase-mimicking Cascade catalysis for ultrasensitive colorimetric detection of Profenofos. Food Chem..

[27] Mekonnen M.L., Abda E.M., Csaki A., Fritzsche W. (2025). Frontiers in laccase nanozymes-enabled colorimetric sensing: A review. Anal. Chim. Acta.

[28] Alizadeh Sani M., Priyadarshi R., Zhang W., Khezerlou A., Rhim J.-W. (2024). Innovative application of laccase enzyme in food packaging. Trends Food Sci. Technol..

[29] Liu Y., Liu L., Qu Z., Yu L., Sun Y. (2023). Supramolecular assembly of benzophenone alanine and copper presents high laccase-like activity for the degradation of phenolic pollutants. J. Hazard. Mater..

[30] Lu Z., Yue J., Lei Q., Sun M., Li X., Li J., Zhu J., Sun D., Li P. (2025). Degradation of AFB(1) in edible oil by amphipathic flower-like immobilized self-assembled peptide-based enzyme mimics. Food Chem..

[31] Huang S., Chen X., Lei Y., Zhao W., Yan J., Sun J. (2022). Ionic liquid enhanced fabrication of small-size BSA-Cu laccase mimicking nanozymes for efficient degradation of phenolic compounds. J. Mol. Liq..

[32] Ren Z., Li X., Zhou M., Jin C., Wang Y., Wang Y., Jiang Y., Liu Y., Huang Z., Sheng D. (2026). A corn protein-based copper nanozyme with laccase-mimicking activity for bisphenol A detection and plastic food packaging degradation. Food Chem..

[33] Li D., Yin J., Yu Z., Gao Z., Xu N., Meng L. (2025). Artificial intelligence-assisted colorimetric sensor array based on supramolecular self-assembled nanozymes for visual monitoring of pesticide residues. Sens. Actuators B Chem..

[34] Makam P., Yamijala S., Bhadram V.S., Shimon L.J.W., Wong B.M., Gazit E. (2022). Single amino acid bionanozyme for environmental remediation. Nat. Commun..

[35] Huang H., Lei L., Bai J., Zhang L., Song D., Zhao J., Li J., Li Y. (2021). Efficient elimination and detection of phenolic compounds in juice using laccase mimicking nanozymes. Chin. J. Chem. Eng..

[36] Liang H., Lin F., Zhang Z., Liu B., Jiang S., Yuan Q., Liu J. (2017). Multicopper Laccase Mimicking Nanozymes with Nucleotides as Ligands. ACS Appl. Mater. Interfaces.

[37] Huang H., Li M., Hao M., Yu L.L., Li Y. (2021). A novel selective detection method for sulfide in food systems based on the GMP-Cu nanozyme with laccase activity. Talanta.

[38] Li J., Cheng Q., Huang H., Li M., Yan S., Li Y., Chang Z. (2020). Sensitive chemical sensor array based on nanozymes for discrimination of metal ions and teas. Luminescence.

[39] Jin P., Zhen L., Zhang X., Bi Z., Li J., Li Y., Huang H. (2025). Machine learning assisted colorimetric sensor array for year and adulteration identification of white peony tea. Microchem. J..

[40] Zhen L., Li J., Bi Z., Zhang X., Jin P., Huang H., Li Y. (2025). A three-channel nanozyme sensor array combined with machine learning algorithm for the differentiation and detection of aminoglycoside antibiotics. Chem. Eng. J..

[41] Xu S., Wu H., Liu S., Du P., Wang H., Yang H., Xu W., Chen S., Song L., Li J. (2023). A supramolecular metalloenzyme possessing robust oxidase-mimetic catalytic function. Nat. Commun..

[42] Wang J., Huang R., Qi W., Su R., Binks B.P., He Z. (2019). Construction of a bioinspired laccase-mimicking nanozyme for the degradation and detection of phenolic pollutants. Appl. Catal. B Environ..

[43] Wang J., Huang R., Qi W., Su R., He Z. (2022). Construction of biomimetic nanozyme with high laccase- and catecholase-like activity for oxidation and detection of phenolic compounds. J. Hazard. Mater..

[44] Fu Z., Guo F., Qiu J., Zhang R., Wang M., Wang L. (2022). Extension of the alkyl chain length to adjust the properties of laccase-mimicking MOFs for phenolic detection and discrimination. Spectrochim. Acta A Mol. Biomol. Spectrosc..

[45] Zhang Y., Zhang K., Yao L., Dong J., Li P., Wang Y., Daka Z., Zheng Y., Liu W., Ji S. (2025). One-step construction of bioinspired multi-enzyme mimicking nanozyme as a universal platform for multi-mode sensing and catalytic degradation. Biosens. Bioelectron..

[46] Song D., Zou Y., Tian T., Ma Y., Huang H., Li Y. (2024). Machine learning-assisted melamine-Cu nanozyme and cholinesterase integrated array for multi-category pesticide intelligent recognition. Biosens. Bioelectron..

[47] Li M., Xie Y., Lei L., Huang H., Li Y. (2022). Colorimetric logic gate for protamine and trypsin based on the Bpy-Cu nanozyme with laccase-like activity. Sens. Actuators B Chem..

[48] Song D., Lei L., Tian T., Yang X., Wang L., Li Y., Huang H. (2023). A novel strategy for identification of pesticides in different categories by concentration-independent model based on a nanozyme with multienzyme-like activities. Biosens. Bioelectron..

[49] Wang L., Li J., Lei L., Li Y., Huang H. (2024). Modulation of the enzyme-like activity of CuAsp nanozyme by gallic acid and the selective detection of bisphenol A in infant food packaging. Anal. Methods.

[50] Jing W., Yang Y., Shi Q., Xu J., Xing G., Dai Y., Liu F. (2024). Nanozymes sensor array for discrimination and intelligent sensing of phenolic acids in food. Food Chem..

[51] Jing W., Yang Y., Shi Q., Wang Y., Liu F. (2024). Machine Learning-Based Nanozyme Sensor Array as an Electronic Tongue for the Discrimination of Endogenous Phenolic Compounds in Food. Anal. Chem..

[52] Ren Z., Deng Q., Wang Y., Yang Y., Wang H., Liu F., Jing W. (2025). Machine learning assisted nanozyme sensor array for accurate identification and discrimination of flavonoids in healthy tea. Food Chem..

[53] Li M., Xie Y., Zhang J., Lei L., Su X. (2023). Construction of a laccase mimic enzyme with fluorescence properties for kanamycin multi-mode analysis. Chem. Eng. J..

[54] Liu S., Yu H., Zhu S., Zhao X.E. (2025). Copper-based fluorescent nanozyme used to construct a ratiometric sensor for visual detection of thiophanate methyl. Talanta.

[55] Zhang X., Wu D., Wu Y., Li G. (2021). Bioinspired nanozyme for portable immunoassay of allergenic proteins based on A smartphone. Biosens. Bioelectron..

[56] Li A., Li H., Ma Y., Wang T., Liu X., Wang C., Liu F., Sun P., Yan X., Lu G. (2022). Bioinspired laccase-mimicking catalyst for on-site monitoring of thiram in paper-based colorimetric platform. Biosens. Bioelectron..

[57] Li J., Wang L., Ma Y., Bi Z., Li Y., Huang H. (2024). Modified Metal-Organic Framework (MOF-818) inspired by natural enzymes for intelligent detection of total antioxidants and bisphenol A in infant beverages. Anal. Chim. Acta.

[58] Yan X., Li H., Wang T., Li A., Zhu C., Lu G. (2022). Engineering of coordination environment in bioinspired laccase-mimicking catalysts for monitoring of pesticide poisoning. Chem. Eng. J..

[59] Li H.H., Murugesan A., Shoaib M., Sheng W., Chen Q.S. (2025). Functionalized metal-organic frameworks with biomolecules for sensing and detection applications of food contaminants. Crit. Rev. Food Sci. Nutr..

[60] Zhao Z., Huang Y., Chao M., Du S., Li J., Wang J., Ma G., Liu Y., Kang X., Fu M. (2025). Oxygen defect engineering CuO@Pd nanocages sensor array for identification of phenolic antioxidants. Chem. Eng. J..

[61] Wang L., Wang B., Kang K., Ji X., Niu Y., Li C., Wang Y. (2025). Defect-engineered porous carbon stabilizes Pt-N4 sites for enhanced laccase-like activity: A nanozyme sensor for sensitive detection of luteolin. Microchem. J..

[62] Liu H., Di Z., Tian Q., Zhou Y., Yang R., Bai Z., Wang H., Chen Y., Zhang L. (2026). Lewis Pair-Engineered CuMnO(x) as Cold-Adapted Multinanozyme for Cooperative Hydrolytic and Oxidative Degradation of Raw Corn Stalk. Adv. Sci..

[63] Liu C., Liu N., Xia Q., Cao Y., Huang Q. (2025). Colorimetric sensor arrays constructed by novel LTP-prepared amorphous Co_3_O_4_ nanozymes with high laccase-like activity for the intelligent identification of phenolic compounds in food. Food Chem..

[64] Wu S.T., Qiu Z.Y., Su H.Q., Cao Y., Gao S.Q., Wang H., Wang C.H., Lin Y.W. (2024). Design of Mn-based nanozymes with multiple enzyme-like activities for identification/quantification of glyphosate and green transformation of organophosphorus. Biosens. Bioelectron..

[65] Li X., Chen M., Li Q., Dai J., Li H., Yang Y. (2026). Green CuNA@Arg-Ce BioMOF with multi enzyme-like catalytic activities for self-cascade one-step colorimetric detection of zearalenone. Food Chem..

[66] Yang W., He H., Lu T., Xiang J., Xu X., Song Y., Tao H., Wu Y. (2026). Wet-chemical synthesis of AgCo@CQAS nanozyme: A novel high-activity copper-free laccase mimic for sensitive hydrogel-based pesticide detection. Biosens. Bioelectron..

[67] Xiao F., Xia Q., Zhang S., Li Q., Chen D., Li H., Yang D., Yang Y. (2024). Ultrasound and defect engineering-enhanced nanozyme with high laccase-like activity for oxidation and detection of phenolic compounds and adrenaline. J. Hazard. Mater..

[68] Zhang J., Li H., Li Y., Han Z., Fu T., Li Y., Wang L., Liu J. (2026). Moderate d-band center determines the high activity of Cu(2)O laccase nanozymes. J. Colloid Interface Sci..

[69] Qi X., Lu J., Li Y., Xu X. (2025). The interfacial optimization and p-n heterojunction construction in nanozyme for improved laccase-mimic and antibiofouling activities. J. Environ. Chem. Eng..

[70] Zhang Z., Zhang Y., Jayan H., Gao S., Zhou R., Yosri N., Zou X., Guo Z. (2024). Recent and emerging trends of metal-organic frameworks (MOFs)-based sensors for detecting food contaminants: A critical and comprehensive review. Food Chem..

[71] Zhang Z.P., Zhou R.Y., Ke L.J., Li J.B., Jayan H., El-Seedi H.R., Zou X.B., Guo Z.M. (2024). Development of multifunctional metal-organic frameworks (MOFs)-based nanofiller materials in food packaging: A comprehensive review. Trends Food Sci. Technol..

[72] Liu S.S., Zhang M.M., Chen Q.S., Ouyang Q. (2024). Multifunctional Metal-Organic Frameworks Driven Three-Dimensional Folded Paper-Based Microfluidic Analysis Device for Chlorpyrifos Detection. J. Agric. Food Chem..

[73] Wang J., Huang R., Qi W., Su R., He Z. (2022). Preparation of amorphous MOF based biomimetic nanozyme with high laccase- and catecholase-like activity for the degradation and detection of phenolic compounds. Chem. Eng. J..

[74] Shams S., Ahmad W., Memon A.H., Wei Y., Yuan Q., Liang H. (2019). Facile synthesis of laccase mimic Cu/H(3)BTC MOF for efficient dye degradation and detection of phenolic pollutants. RSC Adv..

[75] Xu J., Ren Z., Wang Y., Liu F., Jing W. (2025). Histidine-engineered Cu-BTC nanozyme with enhanced laccase-like activity combining the machine learning for precise recognition of Beta-lactam antibiotics. Biosens. Bioelectron..

[76] Xu J., Wang Y., Li Z., Liu F., Jing W. (2025). Machine learning assisted multi-signal nanozyme sensor array for the antioxidant phenolic compounds intelligent recognition. Food Chem..

[77] Geng L., Sun X., Wang L., Liu F., Hu S., Zhao S., Ye F. (2024). Analyte-induced laccase-mimicking activity inhibition and conductivity enhancement of electroactive nanozymes for ratiometric electrochemical detection of thiram. J. Hazard. Mater..

[78] Chen H.L., Wang J.L., Zhang W.W., Li Y.H., Zhang X.N., Huang X.W., Shi Y.Q., Zou Y.C., Li Z.H., Shi J.Y. (2025). Highly catalytic Ce-based MOF for powering electrochemical aptasensing toward evaluating dissolution rate of microelement copper from tea-leaves. J. Food Compos. Anal..

[79] Liang S., Wu X.-L., Xiong J., Yuan X., Liu S.-L., Zong M.-H., Lou W.-Y. (2022). Multivalent Ce-MOFs as biomimetic laccase nanozyme for environmental remediation. Chem. Eng. J..

[80] Liang S., Liu H., Du X., Zhang R., Zhang M., Zong M.H., Lou W.Y. (2025). Bioinspired oxidase nanozymes for chlorpyrifos sensing and tetracyclines eliminating. J. Hazard. Mater..

[81] Ran H., Tang Y., Wu Z., Zhou J., Tao H., Wu Y. (2024). A colorimetric sensor array based on bimetallic CeCo-MOF with triple-enzyme-mimic activities for highly sensitive detection of tetracycline antibiotics. Chem. Eng. J..

[82] Wei W., Wang H., Su P., Song J., Yang Y. (2025). Bioinspired Cu/Zn-ZIF nanozyme with excellent laccase-like activity for selective colorimetric detection of phenolic pollutants. Talanta.

[83] Zou R., Shi J., Lu Q., Sun C., Ye H., Yan X., Tian F., Li H. (2025). Cobalt MOF-hybridized nanozyme catalysts breaking pH limitations for boosted chlorpyrifos sensing performance. Food Chem..

[84] Han E., Gao T., Wang T.Y., Bao M., Cai J.R. (2025). Synergy of yttrium single atom doped carbon nitride and nickel/cobalt spinel ferrites in electrochemical sensing: A novel sensor for simultaneous detection of antioxidants. J. Food Compos. Anal..

[85] Niu X., Wu L., Wu F., Guan J., Wang H. (2023). Electron coupling effect-triggered monatomic copper laccase-mimicking nanozyme for the degradation and detection of guaiacol produced by *Alicyclobacillus acidoterrestris*. Biosens. Bioelectron..

[86] Ma Y., Li Q., Qiu X., Li Z. (2025). Laccase-Inspired Single-Atom Fe-N(4) Nanozymes for Pollutant Degradation and Lignin Valorization. Langmuir.

[87] Qiao C., Wang X., Luo H., Ma Y., Yang P., Huo D., Zhang S., Hou C. (2025). From discoloration to color enhancement: A novel approach to high-concentration tannic acid detection based on VCo-N-C bimetallic Nanozyme. Food Chem..

[88] Sun Y., Wang C., Li H., Wang K., Bai Q., Zhang G., Feng S., Wang L., Zhu Z., Sui N. (2025). sp Carbon Disrupting Axial Symmetry of Local Electric Field for Biomimetic Construction of Three-Dimensional Geometric and Electronic Structure in Nanozyme for Sensing and Microplastic Degradation. Angew. Chem. Int. Ed. Engl..

[89] Wang R., Ma X., Hamed E.M., Cao B., Wang L., Li S.F.Y., Zhu Y. (2025). Deciphering of laccase-like activity ruthenium single-atom nanozyme for identification/quantification and remediation of phenolic pollutants. Sens. Actuators B Chem..

[90] Wang Y., Tian S., Chen S., Li M., Tang D. (2025). S-Modified MOF Nanozyme Cascade System with Multi-Enzyme Activity for Dual-Mode Antibiotic Assay. Anal. Chem..

[91] Huang L., Tang Y., Wang J., Niu X., Zhou J., Wu Y. (2023). Cubic Ag2O nanoparticles as robust laccase mimetics in a smartphone-assisted colorimetric sensor for rapid and ultrasensitive detection of kanamycin in environment. Sens. Actuators B Chem..

[92] Huang L., Tang Y., Han J., Niu X., Lin X., Wu Y. (2024). A stable colorimetric biosensor for highly selective detection of malathion residue in food based on aptamer-regulated laccase-mimic activity. Food Chem..

[93] Xia L., Zheng J., Su J., Yang H., Xie Z., Tang Y., Wu Y. (2025). Portable paper-based sheet for visual detection of ethyl carbamate in Chinese Baijiu based on aptamer recognition and laccase-like activity of cubic Ag_2_O nanoparticles. Food Biosci..

[94] Zhou Y., Wang J., Meng W., Xia L., Tang Y., Zhou J., Wu Y., Yan P. (2026). Smartphone-assisted colorimetric aptasensor for detecting acetamiprid residue in food based on the enhanced laccase-mimic activity of spherical Ag_2_O nanoparticles. Microchem. J..

[95] Lin H., Wang F.Y., Lin J.J., Yang W.J., Kang W.C., Jiang H., Adade S., Cai J.R., Xue Z.L., Chen Q.S. (2023). Detection of wheat toxigenic Aspergillus flavus based on nano-composite colorimetric sensing technology. Food Chem..

[96] Zhang X.A., Zhou Y., Huang X.Y., Hu X.T., Huang X.W., Yin L.M., Huang Q.L., Wen Y.B., Li B., Shi J.Y. (2023). Switchable aptamer-fueled colorimetric sensing toward agricultural fipronil exposure sensitized with affiliative metal-organic framework. Food Chem..

[97] Gao S.P., Xu X.H., Zheng X.Y., Zhang Y., Zhang X.N. (2025). Bimetallic Gold-Platinum (AuPt) Nanozymes: Recent Advances in Synthesis and Applications for Food Safety Monitoring. Foods.

[98] Zhu W.R., Li L.B., Zhou Z., Yang X.D., Hao N., Guo Y.S., Wang K. (2020). A colorimetric biosensor for simultaneous ochratoxin A and aflatoxins B1 detection in agricultural products. Food Chem..

[99] Marimuthu M., Arumugam S.S., Sabarinathan D., Li H.H., Chen Q.S. (2021). Metal organic framework based fluorescence sensor for detection of antibiotics. Trends Food Sci. Technol..

[100] Sharma A.S., Ali S., Sabarinathan D., Murugavelu M., Li H., Chen Q. (2021). Recent progress on graphene quantum dots-based fluorescence sensors for food safety and quality assessment applications. Compr. Rev. Food Sci. Food Saf..

[101] Song D., Tian T., Wang L., Zou Y., Zhao L., Xiao J., Huang H., Li Y. (2024). Multi-signal sensor array based on a fluorescent nanozyme for broad-spectrum screening of pesticides. Chem. Eng. J..

[102] Wang H., Jia C., Huang S., Li S., Liu X., Chen W., Shen Y. (2025). A molecular imprinting-switchable laccase-like nanozyme-based onsite self-calibrating assay of ethyl carbamate residues. Food Chem..

[103] Li W.T., Shi Y.Q., Hu X.T., Li Z.H., Huang X.W., Holmes M., Gong Y.Y., Shi J.Y., Zou X.B. (2019). Visual detection of nitrite in sausage based on a ratiometric fluorescent system. Food Control.

[104] Murugesan A., Li H.H., Shoaib M. (2025). Recent Advances in Functionalized Carbon Quantum Dots Integrated with Metal-Organic Frameworks: Emerging Platforms for Sensing and Food Safety Applications. Foods.

[105] Shoaib M., Li H.H., Khan I.M., Zareef M., Hassan M.M., Niazi S., Chen Q.S. (2024). Emerging MXenes-based aptasensors: A paradigm shift in food safety detection. Trends Food Sci. Technol..

[106] Li Y., Dong Y., Wang R., Lin Z., Lin J., Ji X., Ye B.C. (2024). Biomimetic Electrochemical Sensor Based on Single-Atom Nickel Laccase Nanoenzyme for Quercetin Detection. Anal. Chem..

[107] Wu X.X., Yuan Z.C., Gao S.J., Zhang X.N., El-Mesery H.S., Lu W.J., Dai X.L., Xu R.J. (2025). Electrochemical Biosensors Driving Model Transformation for Food Testing. Foods.

[108] Li C., Zhang X.N., Tang Q.Y., Guo Y.Q., Zhang Z., Zhang W., Zou X.B., Sun Z.B. (2024). Molecularly imprinted electrochemical sensor for ethyl carbamate detection in Baijiu based on "on-off" nanozyme-catalyzing process. Food Chem..

[109] Qu L., Zhang X., Chu Y.H., Zhang Y.Y., Lin Z.Y., Kong F.Z., Ni X., Zhao Y.N., Lu Q.Y., Zou B. (2025). Research Progress on Nanotechnology-Driven Enzyme Biosensors for Electrochemical Detection of Biological Pollution and Food Contaminants. Foods.

[110] Liu S.S., Rong Y.N., Chen Q.S., Ouyang Q. (2024). Colorimetric sensor array combined with chemometric methods for the assessment of aroma produced during the drying of tencha. Food Chem..

[111] Wang Y., Cai J.Z., Lin H., Ouyang Q., Liu Z.H. (2025). Deep learning-assisted self-cleaning cellulose colorimetric sensor array for monitoring black tea withering dynamics. Food Chem..

[112] Huang B.C., Zhang K.X., Kwadzokpui B.A., Han E., Guan B.B., Lin H. (2025). Real-Time Monitoring and Rapid Determination of Pork Freshness Using a Colorimetric Sensor Array Based on Porphyrin and pH Indicator Ligand Combination. J. Agric. Food Chem..

[113] Lai J.J., Ding L.J., Liu Y., Fan C.H., You F.H., Wei J., Qian J., Wang K. (2023). A miniaturized organic photoelectrochemical transistor aptasensor based on nanorod arrays toward high-sensitive T-2 toxin detection in milk samples. Food Chem..

[114] Song D., Tian T., Yang X., Wang L., Sun Y., Li Y., Huang H. (2023). Smartphone-assisted sensor array constructed by copper-based laccase-like nanozymes for specific identification and discrimination of organophosphorus pesticides. Food Chem..

[115] Wang Y., Xu J., Ren Z., Li Y., Liu D., Liu F., Jing W. (2025). Bridging nanozyme sensor array and machine learning for accurate multi-category antibiotics intelligent identification. Chem. Eng. J..

[116] Shi Q., Li Z., Wang Y., Liu F., Jing W. (2025). Machine Learning-Enhanced Nanozyme Sensor Array for Accurate Multiple Quinolone Antibiotics Recognition. Anal. Chem..

[117] Tian T., Song D., Zhang L., Huang H., Li Y. (2024). Facile and selective recognition of sulfonylurea pesticides based on the multienzyme-like activities enhancement of nanozymes combining sensor array. J. Hazard. Mater..

[118] Jing W., Wang Y., Shi Q., Yang Y., Dai Y., Liu F. (2024). Cu(2)(OH)(3)NO(3) nanozyme sensor array for the discrimination of multiple sulfides in food. Biosens. Bioelectron..

[119] Song D., Cheng Y., Hu Y., Ge K., Fan J., Shi X., Huang H., Li Y. (2026). Machine learning-assisted multi-channel nanozyme sensor arrays for multiple pesticide tracking, tracing and metabolism analysis. Biosens. Bioelectron..

[120] Li J., Han Q., Zhang X., Zhen L., Huang H., Li Y. (2026). Novel nanozyme sensor array based on four different enzyme-like activities for the identification of multi-category pesticides. J. Hazard. Mater..

[121] Wu G., Du C., Peng C., Qiu Z., Li S., Chen W., Qiu H., Zheng Z., Lu Z., Shen Y. (2024). Machine learning-assisted laccase-like activity nanozyme for intelligently onsite real-time and dynamic analysis of pyrethroid pesticides. J. Hazard. Mater..

[122] Chen X.D., Zhao C.L., Zhao Q.W., Yang Y.F., Yang S.X., Zhang R.M., Wang Y.Q., Wang K., Qian J., Long L.L. (2024). Construction of a Colorimetric and Near-Infrared Ratiometric Fluorescent Sensor and Portable Sensing System for On-Site Quantitative Measurement of Sulfite in Food. Foods.

[123] Zhang C., Zhou Y.F., Ming L., Chen L.H., Xue M.Q., Zhang J., Zhang H.Y. (2025). Dual-mode strategy for the determination of vanillin in milk-based products based on molecular-imprinted nanozymes. Food Chem..

[124] Arshad A., Ding L.J., Akram R., Zhu W.R., Long L.L., Wang K. (2025). Construction of a novel Au@Os mediated TMB-H_2_O_2_ platform with dual-signal output for rapid and accurate detection of ziram in food. Food Chem..

[125] Hu X.T., Shi J.Y., Shi Y.Q., Zou X.B., Tahir H.E., Holmes M., Zhang W., Huang X.W., Li Z.H., Xu Y.W. (2019). A dual-mode sensor for colorimetric and fluorescent detection of nitrite in hams based on carbon dots-neutral red system. Meat Sci..

[126] Li W.T., Zhang X.A., Shi Y.Q., Hu X.T., Wang X., Liang N.N., Shen T.T., Zou X.B., Shi J.Y. (2023). A dual-modal biosensor coupling cooperative catalysis strategy for sensitive detection of AFB1 in agri-products. Food Chem..

[127] Liang N.N., Hu X.T., Li W.T., Mwakosya A.W., Guo Z., Xu Y.W., Huang X.W., Li Z.H., Zhang X.A., Zou X.B. (2021). Fluorescence and colorimetric dual-mode sensor for visual detection of malathion in cabbage based on carbon quantum dots and gold nanoparticles. Food Chem..

[128] Yin L.M., Cai J.R., Ma L.X., You T.Y., Arslan M., Jayan H., Zou X.B., Gong Y.Y. (2024). Dual function of magnetic nanocomposites-based SERS lateral flow strip for simultaneous detection of aflatoxin B1 and zearalenone. Food Chem..

[129] Lin H., Zhang K.X., Guo J.L., Kwadzokpui B.A., Adade S., Chen Q.S. (2024). Olfactory analysis of oolong tea sensory quality using composite nano-colorimetric sensor array. Food Res. Int..

[130] Salah M., Sobhy M., Fang Y.J., El-Sayed S.M., Khalifa I., Korin A., Mansour M., Bacha S.A.S., Maqsood S., Wang Y. (2025). Development and characterization of BSA-pullulan gel matrices incorporating orange peel flavonoids: Structural interactions and antimicrobial and antioxidant functionalities. Food Chem..

[131] He Y.F., Liu T., Larsen D.S., Lei Y.X., Huang M.C., Zhu L., Daglia M., Xiao X. (2025). Barley fermentation on nutritional constituents: Structural changes and structure-function correlations. Crit. Rev. Food Sci. Nutr..

[132] He Y.F., Chen M.Z., Tang Y.X., Tui W., Gan Q., Zhu Y., Zhang J.Y., Zhao Y.S., Zhang X.P., Xiao X. (2026). Fermented barley bran addition improved the digestive properties, gastrointestinal phenol release and the antioxidant activities of extruded rice. Food Funct..

[133] Arslan M., Zareef M., Afzal M., Tahir H.E., Shi J.Y., Muhammad A., Rakha A., Xiaobo Z. (2025). A smart olfactory visualization system based on colorimetric sensor array and chemometrics for the identification of rice adulteration. Food Chem..

[134] Bilal M., Arslan M., Samee U., Shishir M.R.I., Shaukat F., Li Z.H., Xia S., Xiaobo Z. (2025). Efficient detection of adulteration in peanut seed oil using a smartphone-based colorimetric sensor array system. J. Food Compos. Anal..

[135] Zhai X.D., Xue Y.H., Sun Y., Ma X.D., Ban W.W., Marappan G., Tahir H.E., Huang X.W., Wu K.L., Chen Z.L. (2025). Colorimetric Food Freshness Indicators for Intelligent Packaging: Progress, Shortcomings, and Promising Solutions. Foods.

[136] Kang W.C., Lin H., Jiang H., Adade S., Xue Z.L., Chen Q.S. (2021). Advanced applications of chemo-responsive dyes based odor imaging technology for fast sensing food quality and safety: A review. Compr. Rev. Food Sci. Food Saf..

[137] Zhang X.R., Huang X.Y., Dai C.X., Tian X.Y., Wang C.Q., Ren Y., Wang L., Yu S.S., Aheto J.H., Chang X.H. (2025). Characterization of volatile flavor profiles and quantitative assessment of key physicochemical indicators for fermented bean curd using GC-IMS, E-nose and multi-channel colorimetric sensor array combined with chemometrics. Food Biosci..

[138] Zhang X.R., Huang X.Y., Aheto J.H., Ren Y., Wang L., Yu S.S., Wang Y. (2024). Comparable analysis of flavor compounds and quality assessment of fermented bean curd using HS-SPME-GC/MS and colorimetric sensor array. Food Biosci..

[139] Okoye C.O., Jiang H.F., Ezenwanne B.C., Jiang J.X. (2025). Advances in tea processing and mycotoxin detection: Prospects of machine learning-assisted multi-omics for enhancing safety and quality. J. Food Compos. Anal..

[140] Murugesan A., Li H.H. (2026). Toward Sustainable Food Packaging: Innovations in Biodegradable Materials, Smart Technologies, and AI Integration. Compr. Rev. Food Sci. Food Saf..

[141] Pan Y., Wang W., Dai C., Zhou M., Zhang L., Sun L., Cai M., He R. (2026). Bacillus-Derived Lipopeptides as Green Biosurfactants: Structures, Functions, Production, and Safety for Sustainable Applications. Compr. Rev. Food Sci. Food Saf..

[142] Zhao X.C., Zhang J.Y., Lu C.X., Zi Y.C., Xiao X. (2025). Types of polyphenolic compounds in cereals and their microbial transformation. Food Biosci..

[143] Wang B., Niu Y., Wang L., Shen Y., Wang L., Li X., Li D., Kang K., Ji X. (2026). Iron single-atom nanozyme-enabled dual-mode electrochemical and colorimetric sensing for ultrasensitive detection of chlorogenic acid. Microchem. J..

[144] Li X., Yan J., Zhang Y., Wang Y. (2025). Bifunctional Mn-polysaccharide nanozyme: Smartphone-enabled tri-detection and efficient catechol/phenol degradation. Microchem. J..

[145] Davoodi-Rad K., Shokrollahi A., Shahdost-Fard F., Azadkish K., Madani-Nejad E. (2024). A smartphone-based colorimetric assay using Cu-tannic acid nanosheets (Cu-TA NShs) as a laccase-mimicking nanozyme for visual detection of quercetin in vegetables. Mikrochim. Acta.

[146] Liu D., Yang J., Wen S., Mu M., Ji W., Zhao B., Ozaki Y., Song W. (2024). Multienzyme-like MnO_2_@Ag aerogel as SERS/colorimetric sensing platform for the fermentation process of tea and assess the total antioxidant capacity. Sens. Actuators B Chem..

[147] Liu C., Liu N., Xia Q., Huang Q. (2026). MnZn bimetallic cold-adapted nanozymes prepared by DBD plasma for in situ monitoring of volatile sulfur compounds and meat freshness inspection. Food Chem..

[148] Dzah C.S., Duan Y.Q., Zhang H.H., Boateng N.A.S., Ma H.L. (2020). Latest developments in polyphenol recovery and purification from plant by-products: A review. Trends Food Sci. Technol..

[149] Khan K.Y., Ali B., Li G.L., Iqbal B., Siddiqui N.R., Jabbar S., Ali I., Hassan A.M., Binjawhar D.N., Abdel-Hameed U.K. (2024). Distribution of nutrients, bioactive compounds, and antioxidant properties of grain-based milling fractions of *Glycine max* L.. Cyta-J. Food.

[150] Tang Y.X., Liu W.J., Zhang J.Y., Juan B., Zhu Y., Zhu L., Zhao Y.S., Daglia M., Xiao X., He Y.F. (2025). Advances in Intestinal-Targeted Release of Phenolic Compounds. Nutrients.

[151] Dzah C.S., Duan Y.Q., Zhang H.H., Ma H.L. (2021). Effects of pretreatment and type of hydrolysis on the composition, antioxidant potential and HepG2 cytotoxicity of bound polyphenols from Tartary buckwheat (*Fagopyrum tataricum* L. *Gaerth*) hulls. Food Res. Int..

[152] Li F.N., Boateng I.D., Chen S.M., Yang X.M., Soetanto D.A., Liu W.M. (2023). Pulsed light irradiation improves degradation of ginkgolic acids and retainment of ginkgo flavonoids and terpene trilactones in *Ginkgo biloba* leaves. Ind. Crops Prod..

[153] Boateng I.D., Li F.N., Yang X.M., Guo D.Z. (2024). Combinative effect of pulsed-light irradiation and solid-state fermentation on ginkgolic acids, ginkgols, ginkgolides, bilobalide, flavonoids, product quality and sensory assessment of *Ginkgo biloba* dark tea. Food Chem..

[154] Mamy D., Boateng I.D., Chen X.M. (2025). Metabolomic changes in Citrus reticulata peel after conventional and ultrasound-assisted solid-state fermentation with Aspergillusniger: A focus on flavonoid metabolism. Food Chem..

[155] Wang F., Wang M., Wang M., Xu L., Qian J., Guan G., Xu B. (2025). Clarification of Sugarcane Juice Catalyzed by Magnetic Immobilized Laccase Intensified by Alternating Magnetic Field. Foods.

[156] Liang Y., Lin H., Kang W.C., Shao X.K., Cai J.R., Li H.H., Chen Q.S. (2023). Application of colorimetric sensor array coupled with machine-learning approaches for the discrimination of grains based on freshness. J. Sci. Food Agric..

[157] Pan Y., Dai C., Zhang L., Zhou M., Zhu S., Huang L., He R. (2026). Application of Ultrasonication as an Emerging Non-Thermal Physical Technology in Meat Product Processing: A Review. Foods.

[158] Zhang J.J., Qin Z., Zhang R.J., Zhang J.N., Huang X.W., Zhang L.D., Shen T.T., Shi J.Y., Zou X.B. (2025). Advances in Controlled-Release Packaging for Food Applications. Compr. Rev. Food Sci. Food Saf..

[159] Han Z., Ahmad W., Rong Y.N., Chen X.Y., Zhao S.G., Yu J.H., Zheng P.F., Huang C.C., Li H.H. (2024). A Gas Sensors Detection System for Real-Time Monitoring of Changes in Volatile Organic Compounds during Oolong Tea Processing. Foods.

[160] Ouyang Q., Rong Y.N., Wu J.Q., Wang Z., Lin H., Chen Q.S. (2023). Application of colorimetric sensor array combined with visible near-infrared spectroscopy for the matcha classification. Food Chem..

[161] Wang Y., Shoaib M., Wang J.Y., Lin H., Chen Q.S., Ouyang Q. (2025). A novel ZIF-8 mediated nanocomposite colorimetric sensor array for rapid identification of matcha grades, validated by density functional theory. J. Food Compos. Anal..

[162] Gao S.P., Shen T.T., Zhang Y. (2026). Photothermal lateral flow assays for food contaminant detection: Principles, nanoprobes, and emerging detection strategies. Food Control.

[163] Gao S.P., Zhou R.Y., Zhang D., Zheng X.Y., El-Seedi H.R., Chen S.Q., Niu L.D., Li X., Guo Z.M., Zou X.B. (2024). Magnetic nanoparticle-based immunosensors and aptasensors for mycotoxin detection in foodstuffs: An update. Compr. Rev. Food Sci. Food Saf..

[164] Wang T.S., Ding L.J., Zhang M., Wang D.P., Zhu W.R., Wang K. (2026). A high-sensitivity magnetic-controlled separable visuable aptasensor based on neutral pH SOD-like dual-metal based MOF on MOF for aflatoxin B1 detection. Food Chem..

[165] Liu Z.J., Wang X.Y., Ren X.X., Li W.B., Sun J.F., Wang X.W., Huang Y.Q., Guo Y.G., Zeng H.W. (2021). Novel fluorescence immunoassay for the detection of zearalenone using HRP-mediated fluorescence quenching of gold-silver bimetallic nanoclusters. Food Chem..

[166] Bi X.Y., Li L.B., Liu X.H., Luo L.J., Cheng Z.L., Sun J.Y., Cai Z.B., Liu J.M., You T.Y. (2021). Inner filter effect-modulated ratiometric fluorescence aptasensor based on competition strategy for zearalenone detection in cereal crops: Using mitoxantrone as quencher of CdTe QDs@SiO_2_. Food Chem..

[167] Zhu C.X., Liu D., Li Y.Y., Chen T., You T.Y. (2022). Label-free ratiometric homogeneous electrochemical aptasensor based on hybridization chain reaction for facile and rapid detection of aflatoxin B1 in cereal crops. Food Chem..

[168] Liu T., He J., Yao W., Jiang H., Chen Q. (2022). Determination of aflatoxin B1 value in corn based on Fourier transform near-infrared spectroscopy: Comparison of optimization effect of characteristic wavelengths. Lwt-Food Sci. Technol..

[169] Shen L.Q., Wang Y.F., Li X., Hou Z.Q., Mao J., Shi J.Y., Battino M., Routledge M.N., Gong Y.Y., Zou X.B. (2024). Spatial-temporal distribution of deoxynivalenol, aflatoxin B1, and zearalenone in the solid-state fermentation basin of traditional vinegar and their potential correlation with microorganisms. Food Chem..

[170] He P.H., Hassan M.M., Yang W.J., Shi Z.X., Zhou X.Y., Xu Y., Ouyang Q., Chen Q.S. (2023). Rapid and stable detection of three main mycotoxins in rice using SERS optimized AgNPs@K30 coupled multivariate calibration. Food Chem..

[171] Wu B., Liu X., Chen C., Wang X. (2024). Iron-copper nanozyme mimicking laccase for colorimetric determination of deoxynivalenol in feed. Microchem. J..

[172] Zhang X., Lv H., Rao Z., Yin J., Zhang W., Zhao L., Wang Z., Guo Y. (2025). Exploring of Cu-tannic acid laccase-mimicking nanozyme for effective degradation of aflatoxin B1. Innov. Food Sci. Emerg. Technol..

[173] Dai C.H., Pan Y., Zhou M., Zhang L.H., Huang L.R., Duan Y.Q., He R.H. (2026). Ultrasound-assisted cleaning and drying in food processing: Mechanisms, applications, and quality enhancements. Food Control.

[174] Okoye C.O., Ezenwanne B.C., Olalowo O.O., Ajanwachukwu O.J., Chukwudozie K.I. (2026). Microbial-mycotoxin interactions in food: A review of ecotoxicological implications and omics approaches for understanding detoxification mechanisms. Food Microbiol..

[175] Ashiagbor K., Jayan H., Yosri N., Amaglo N.K., Zou X.B., Guo Z.M. (2025). Advancements in SERS based systematic evolution of ligands by exponential enrichment for detection of pesticide residues in fruits and vegetables. Food Chem..

[176] Xue Y.C., Jiang H. (2023). Monitoring of Chlorpyrifos Residues in Corn Oil Based on Raman Spectral Deep-Learning Model. Foods.

[177] Yang N., Wang P., Xue C.Y., Sun J., Mao H.P., Oppong P.K. (2018). A portable detection method for organophosphorus and carbamates pesticide residues based on multilayer paper chip. J. Food Process Eng..

[178] Liu Z., Feng Y., Cui Y., Wang Y., Fu Z., Zhao J., Li H., Sun C. (2025). Tuning ligand substituents of metal-organic frameworks enhances laccase-like activity for pesticide detection. Food Chem..

[179] Huang Y., Liu C., Xiong Y., Wen Q., Li W., Ren J., Zhong H. (2025). Discrimination of multiplex pesticides using a multienzyme-like metal-organic framework nanozyme. J. Hazard. Mater..

[180] Xia L., Han J., Huang X., Niu X., Lin X., Wu Y. (2024). Colorimetric sensor and visual enzyme sheets for sensitive detection of dimethoate residue in vegetables based on laccase-like activity of coral-like silver citrate. Food Control.

[181] Duan Y.L., Han W.Y., Guo P., Wei X.H. (2024). YOLOv8-GDCI: Research on the Phytophthora Blight Detection Method of Different Parts of Chili Based on Improved YOLOv8 Model. Agronomy.

[182] Ma J., Zhao Y.K., Fan W.P., Liu J.Z. (2024). An Improved YOLOv8 Model for Lotus Seedpod Instance Segmentation in the Lotus Pond Environment. Agronomy.

[183] Zhou X.Y., Chen W.M., Wei X.H. (2024). Improved Field Obstacle Detection Algorithm Based on YOLOv8. Agriculture.

[184] Wang P.Y., Li H.H., Hassan M.M., Guo Z.M., Zhang Z.Z., Chen Q.S. (2019). Fabricating an Acetylcholinesterase Modulated UCNPs-Cu2+ Fluorescence Biosensor for Ultrasensitive Detection of Organophosphorus Pesticides-Diazinon in Food. J. Agric. Food Chem..

[185] Gao S.P., Yang W.H., Zheng X.Y., Wang T.X., Zhang D., Zou X.B. (2025). Advances of nanobody-based immunosensors for detecting food contaminants. Trends Food Sci. Technol..

[186] Huang R., Zigale T.T., Meng H., Wang L., Dong Q.W., Zeng K., Zhang Z. (2025). Cobalt Single-Atom Nanozyme-Enabled Multimodal Lateral Flow Immunoassay for On-Site Ultrasensitive Detection of Tetracycline Residues in Agri-Food Products. J. Agric. Food Chem..

[187] Gan Z.Y., Hu X.T., Xu X.C., Zhang W., Zou X.B., Shi J.Y., Zheng K.Y., Arslan M. (2021). A portable test strip based on fluorescent europium-based metal-organic framework for rapid and visual detection of tetracycline in food samples. Food Chem..

[188] Wu C., Li J., Song J., Guo H., Bai S., Lu C., Peng H., Wang X. (2024). Novel colorimetric detection of oxytetracycline in foods by copper nanozyme. Food Chem..

[189] Wu S., Niu X., Chen J., Meng W., Tao H., Zhou J., Wu Y. (2025). Design of Mn-Car multivalent laccase mimics through self-assembled coordination and its application for ultrasensitive colorimetric detection of kanamycin in food. Microchem. J..

[190] Zhang J.W., Wang H., Hu J.Y., Liu S., Shen Y.Z. (2026). Smartphone-assisted Colorimetric/Fluorescent Collaborative Sensing Platform Based on Laccase-like Activity Fluorescent Nanozyme for Visual Monitoring of β-Lactam Antibiotics. Chin. J. Anal. Chem..

[191] Ali R., Alharbi A.H., Alatwi M.H., Albalawi F.M., Albalawi E.M., Alahmadi A.H., Alanazi N.M., Humadi A.M., Albalawi A.Z.A., El-Wekil M.M. (2026). Colorimetric detection of kasugamycin via silver oxide nanoparticles with intrinsic laccase activity. Food Chem..

[192] Gutema K.F., Mekonnen M.L., Yilma B.T., Asrat T.E., Dellith J., Diegel M., Csaki A., Fritzsche W. (2025). Rapid Colorimetric Detection of Sulfite in Red Wine Using Alginate-Copper Laccase Nanozyme with Smartphone as an Optical Readout. ACS Meas. Sci. Au.

[193] Li J., Wang L., Song D., Li Y., Huang H. (2024). A nanozyme with switchable enzyme-like activity for the logic gates detection of thymol and hydrogen peroxide in honey. Talanta.

[194] Li M., Xie Y., Song D., Huang H., Li Y. (2023). 2-Methylimidazole-doped nanozymes with enhanced laccase activity for the (+)-catechins detection in dairy products. Talanta.

[195] Wei Y., Huang L., Shi Z., Tang Y., Huang X., Wu Y. (2024). Smartphone-integrated colorimetric sensor for rapid and highly selective detection of spermine in food based on the laccase-mimicking activity of flower-shaped Mn_3_O_4_ nanoparticles. Microchem. J..

[196] Zhou J., Wang J., Huang X., Xia L., Tao H., Wu Y. (2024). Coral-like silver citrate as robust laccase mimetics in a novel colorimetric aptasensor for sensitive and highly selective detection of histamine in food. Food Control.

[197] Wu Z., Wei Y., Tang Y., Xia L., Niu X., Wu Y. (2025). Switching off-on colorimetric sensor for rapid and selective detection of histamine in aquatic products based on Cu^2+^-mediated laccase-mimic activity of Mn_3_O_4_ nanoparticles. Food Control.

[198] Wang L., Chen Y. (2022). High-performance, synergistically catalytic luminescent nanozyme for the degradation and detection of endocrine-disrupting chemical diethylstilbestrol. Mater. Today Chem..

[199] Hu M., Yue Z., Zhao F., Lei Z. (2026). Simple colorimetric detection of Cu2+ in water and alcoholic beverages based on D-penicillamine-mediated laccase-like activity of PDA/MnOx nanoparticles. Food Chem..

[200] Shoaib M., Li H.H., Zareef M., Khan I.M., Iqbal M.W., Niazi S., Raza H., Yan Y.Y., Chen Q.S. (2025). Recent Advances in Food Safety Detection: Split Aptamer-Based Biosensors Development and Potential Applications. J. Agric. Food Chem..

[201] Bilal M., Arslan M., Khan S., Shi J., Li Z., Guo Y., Sun X., Xiaobo Z. (2025). Sustainable food packaging: Life cycle assessment, recycling innovations, and *Pestalotiopsis microspora* in biodegradable solutions. Trends Food Sci. Technol..

